# Influence of Polymeric Dispersants on the Dissolution Rate of Tricalcium Silicate and the Nucleation of Calcium‐Silicate‐Hydrate and Portlandite

**DOI:** 10.1002/chem.202500207

**Published:** 2025-04-25

**Authors:** Andreas Vohburger, Marie Collin, Olivia Rindle, Torben Gädt

**Affiliations:** ^1^ Chair for the Chemistry of Construction Materials TUM School of Natural Sciences Technical University of Munich Garching Germany

**Keywords:** calcium‐silicate‐hydrate, dissolution, nucleation, portland cement, superplasticizers

## Abstract

Polycarboxylate ether (PCE) and polyphosphate ether (PPE) dispersants (also known as superplasticizers) are indispensable components of modern concrete mix designs. They reduce the yield stress of cement suspensions and thereby facilitate the reduction of the water‐to‐cement ratio. However, PCEs and PPEs also cause retardation of the cement hydration as a secondary effect, which causes delayed concrete strength development. It is proposed that the retardation is caused by the polymer influence on the tricalcium silicate dissolution rate and the crystallization of the hydration products (e.g., calcium hydroxide Ca(OH)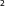 and calcium‐silicate‐hydrate $1.7\mathrm{CaO}\cdot {\mathrm{SiO}}_{2}\cdot 4H_{2}O$ or C‐S‐H). This study examines the effect of four different polymeric superplasticizers with carboxylate and phosphate groups on the reaction kinetics of the pure cement phase tricalcium silicate (in cement shorthand $C_{3}S$). Our results confirm the strong retarding effect of the polymers on the hydration reaction of the $C_{3}S$ paste. Based on model experiments, we studied the polymer influence on the dissolution of $C_{3}S$ and the nucleation of the hydration products. We observed no $C_{3}S$ dissolution‐inhibiting effect of the polymers at the chosen concentrations and undersaturation. However, such an inhibiting effect may occur at solution conditions closer to the saturation limit. Titrimetric analyses indicate that all polymers suppress the nucleation of portlandite and C‐S‐H, with a higher polymer charge density leading to a more pronounced inhibition.

## Graphical abstract

Comb‐copolymers with anionic functional groups and poly(ethylene glycol) side chains are concrete dispersants. However, they retard the hydration of the main Portland cement phase ${\mathrm{Ca}}_{3}S{\mathrm{iO}}_{5}$. Herein, we report on their influence on the dissolution rate of ${\mathrm{Ca}}_{3}S{\mathrm{iO}}_{5}$ and their nucleation‐inhibiting effect on calcium‐silicate‐hydrate and calcium hydroxide. For phosphate and carboxylate groups, higher charge densities lead to stronger nucleation inhibition.

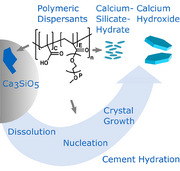

## 1 Introduction

Concrete can be immensely improved with chemical admixtures as minor mix design components in addition to aggregates, cement, and water. Polymeric dispersants, so‐called superplasticizers, are the most important admixture class. These superplasticizers act as cement dispersants and decrease the yield stress by deflocculating the cement particles.^[^ ^1^, ^2^, ^3^, ^4^, ^5^ ^]^ Thereby, superplasticizers enable a reduction of the water‐to‐cement ratio (w/c) in concrete and, therefore, increase the final concrete strength.^[^ ^6^ ^]^ This facilitates a reduction of the cement content in concrete mix designs and, consequently, significantly reduces the carbon footprint of concrete.^[^ ^7^ ^]^ Polycarboxylate ethers (PCEs) are the most prominent superplasticizer class. Structurally, they are comb‐shaped copolymers with a carboxylate group bearing backbone and side chains of poly(ethylene glycol) (PEG).^[^ ^8^ ^]^ Synthetically, these copolymers are commonly obtained by copolymerization of acrylic or methacrylic acid with PEG‐bearing olefinic monomers, such as MPEG‐methacrylates or ethoxylated isoprenol.^[^ ^4^ ^]^ The carboxylate group causes the adsorption of the polymers on the cement grain surface, while the PEG chains impart steric repulsive forces between the cement particles.^[^ ^9^, ^10^, ^11^, ^12^, ^13^ ^]^

In addition to carboxylic groups, phosphate groups have also been explored as anchor groups.^[^ ^4^, ^14^, ^15^, ^16^, ^17^ ^]^ The calcium‐binding affinity of polyphosphate ethers (PPEs) is significantly higher than for PCEs due to the double negatively charged phosphate group in contrast to the single negative charge of the carboxylate group.^[^ ^17^ ^]^ The stronger adsorption of the PPE polymers on the surface of the cement particles decreases the sulfate sensitivity of the superplasticizers.^[^ ^4^, ^16^ ^]^

An undesired side effect of superplasticizers is a retardation of the cement hydration, especially the hydration of the tricalcium silicate phase (${\mathrm{Ca}}_{3}S{\mathrm{iO}}_{5}$ is written as $C_{3}S$ in cement chemistry shorthand, the relevant symbols being C ═ CaO, S ═ ${\mathrm{SiO}}_{2}$, H ═ $H_{2}O$).^[^ ^18^ ^]^ Mechanistically, the hydration of $C_{3}S$ consists of two steps. After contact with water, $C_{3}S$ starts to dissolve, and the ion activity of calcium, silicate, and hydroxide in the pore solution increases (Equation 1)

(1) $${\mathrm{Ca}}_{3}{\mathrm{SiO}}_{5}+5H_{2}O⟶3{\mathrm{Ca}}^{2+}+H_{4}{\mathrm{SiO}}_{4}+6\;{\mathrm{OH}}^{-}$$

The pore solution quickly becomes supersaturated with regard to calcium hydroxide (portlandite, Ca(OH)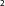 or CH) and calcium‐silicate‐hydrate ($1.7\mathrm{CaO}\cdot {\mathrm{SiO}}_{2}\cdot 4H_{2}O$ or C‐S‐H). Note that the stoichiometry of C‐S‐H is flexible and depends on several parameters, such as the Ca to Si ratio.^[^ ^19^ ^]^ The second step is the nucleation and crystal growth of Ca(OH)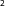 and C‐S‐H that occurs after an induction period and can be described by Equations 2 and 3

(2) $$\begin{array}{cc} {\mathrm{Ca}}^{2+} & +2{\mathrm{OH}}^{-}⟶{\mathrm{Ca}(\mathrm{OH})}_{2}↓ \end{array}$$

(3) $$1.7{\mathrm{Ca}}^{2+}\;+\;H_{2}{{\mathrm{SiO}}_{4}}^{2-}\;+\;1.4{\mathrm{OH}}^{-}+2.3H_{2}O\;\to 1.7\mathrm{CaO}\cdot {\mathrm{SiO}}_{2}\cdot 4H_{2}O\;↓$$

Consequently, the hydration can be described as the sequence of dissolution of the clinker phase and subsequent crystallization of the hydrate phases. Therefore, the hydration reaction rate is determined by the slowest step. The overall hydration reaction of tricalcium silicate can be written as Equation 4 or Equation 5

(4) $$\begin{array}{c} {\mathrm{Ca}}_{3}{\mathrm{SiO}}_{5}+5.3H_{2}O⟶1.3{\mathrm{Ca}(\mathrm{OH})}_{2}+1.7\mathrm{CaO}\cdot {\mathrm{SiO}}_{2}\cdot 4H_{2}O \end{array}$$

(5) $$C_{3}S+5.3H⟶1.3\mathrm{CH}+C_{1.7}{\mathrm{SH}}_{4}$$

PCEs were shown to reduce the dissolution rates of $C_{3}S$ at high concentrations and low undersaturations using a flow‐through setup combined with vertical scanning interferometry.^[^ ^20^, ^21^ ^]^ Furthermore, carboxylic group‐bearing polymers like PCEs also inhibit the nucleation of C‐S‐H and CH.^[^ ^22^, ^23^ ^]^ Therefore, it is firmly established that PCE superplasticizers influence both fundamental reaction steps in the same manner: they retard, i.e., they slow down the total rate of hydration of cement. However, the effect strength for a consistent set of polymers on the dissolution and crystallization step of $C_{3}S$ hydration has not been compared systematically.

This study investigates the effect of two carboxylate and two phosphate‐based superplasticizers with different charge densities. First, we use isothermal heat flow calorimetry to describe the effect of the polymers on the hydration reaction of $C_{3}S$. Then, we separately study each polymer at different dosages to determine their effects on dissolution and crystallization. The dissolution experiments measure the dissolution rates using a $C_{3}S$ suspension with very high water content (to avoid the formation of a supersaturated solution) with a simple ICP‐OES setup.^[^ ^24^ ^]^ The nucleation and crystallization experiments are carried out using an automated titration system to create homogeneous and supersaturated solutions for CH or C‐S‐H.^[^ ^25^, ^26^, ^27^ ^]^ By comparing the magnitude of the effects caused by the different polymers, we strive to clarify whether dissolution or crystallization is the rate‐determining step in cement hydration in the presence of superplasticizers. Additionally, we compare the influence of the anionic groups, i.e., phosphate and carboxylate, on the retardation of $C_{3}S$ hydration and the influence on dissolution and crystallization.

## 2 Materials and Methods

### 2.1 Materials, Polymers, and $C_{3}S$

Unless stated otherwise, all chemicals (the purity is given in parentheses) used in this study were commercially sourced and were used without further purification: ${\mathrm{CaCl}}_{2}\cdot 2H_{2}O$ (99.5%), NaOH (pellets, 99%), ${\mathrm{Na}}_{2}{\mathrm{SiO}}_{3}\cdot 9H_{2}O$ (99%), 3‐mercaptopropionic acid (3‐MPA), methacrylic acid (MA) (99 %), polyphosphoric acid (for synthesis, 83%to87%), methoxy polyethylene glycol 5000‐methacrylate (MPEG5000‐MA), and sodium peroxodisulfate (NaPS).

**Figure 1:**
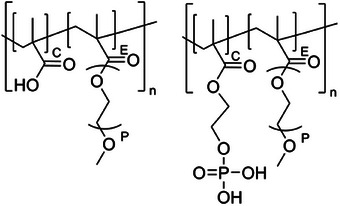
Molecular structure of PCE and PPE polymers.

MPEG5000‐MA was purified by ion exchange to remove methacrylic acid using an ion exchange resin (Dowex Marathon TH 4200 Cl Ion, see S1.1). All solutions were prepared using degassed water to avoid carbonate contamination. Due to the higher ${\mathrm{CO}}_{2}$ affinity of alkaline solutions, the NaOH and silicate solutions were prepared daily. The degassing was done by heating ultrapure water (NANOpure Diamond, Barnstead) to a boil for 20 minutes followed by sparging with nitrogen for another 20 minutes. The synthesis of the phosphate based monomer (2‐(methacryloyloxy) ethyl phosphate, MHP), the polymers and the tricalcium silicate is described in detail in the Supporting Information sections S1.3, S1.4, and S1.5. The polymer characterization is found in Section S1.4 and is summarized in Table 1. The molecular structures of the different types of polymers are shown in Figure 1.

**Table 1:** Properties of the synthesized polymers. For all polymers, MPEG5000‐MA was used as macromonomer (E) with a side chain length (P) of approx. *P* = 113 with a mass of $5000\;g\;{\mathrm{mol}}^{-1}$.

Polymer	Monomer (C)	C/E Ratio Targeted	C/E Ratio Measured	Backbone length (n)	$M_{n}$ $\left(g\;{\mathrm{mol}}^{-1}\right)$	$M_{w}$ $\left(g\;{\mathrm{mol}}^{-1}\right)$	PDI
PCE5	MA	5	5.1	7.7	43514	79212	1.82
PCE10	MA	10	10.2	4.8	29193	50241	1.72
PPE2.5	MHP	2.5	2.0	10.1	56790	109960	1.94
PPE5	MHP	5	2.9	8.4	48982	104800	2.14

Gel‐permeation chromatography was used to determine the number average molecular weight $\left(M_{n}\right)$, the weight average molecular weight $\left(M_{w}\right)$, and the polydispersity (PDI). The molar C/E ratio (C: the number of the charge monomer, E: the number of the side‐chain monomer in the repeat unit, nomenclature according to Flatt^[^ ^10^ ^]^), was experimentally determined by titration (see Section S1.4).

The polymer design was based on the initial molar C/E ratio, used for the synthesis. Although there is a good correspondence between the targeted and measured C/E ratio for the PCE‐type polymers, there is a significant deviation for the phosphate‐based ones. Impurities in the phosphate‐based monomer (Figure S1 and Table S1) lead to several byproducts in the polymerization reaction, lowering the C/E ratio of the final product. To make the synthesis simple and scalable, we chose a synthesis route of the phosphate monomer, which involves polyphosphoric acid as a phosphorylating agent. Unfortunately, the formation of byproducts such as the di‐HEMA ester of phosphoric acid or excess phosphoric acid cannot be avoided with this protocol. By‐products were removed by purification (the detailed procedure is shown in Section S1.4). The shown measured C/E ratio was determined for the final polymer after purification.

### 2.2 Dissolution Studies

Solid $C_{3}S$ was dissolved in pure water at a water‐to‐solid ratio (w/s) of 10,000, guaranteeing that the solution remains undersaturated after full dissolution. A 500mL three‐neck flask was equipped with a pH electrode to monitor the pH changes throughout the experiment. The flask was filled with 500mL of degassed, ultra‐pure water. A gentle flow of dry nitrogen was continuously bubbled through the solution to prevent carbonation. The polymer solutions were diluted with water to achieve a total concentration of $4\;g\;L^{-1}$. This concentration was chosen based on similar experiments documented in the literature.^[^ ^20^ ^]^ It represents an additive dosage of 0.2%‐bwoc in cement pastes with a water‐to‐cement ratio of 0.5, falling within the typical dosage range. To lower the undersaturation conditions of the experiments, calcium metal was added to the solutions to achieve an initial Ca(OH)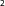 concentration of 6 or $10\;m\mathrm{mol}\;L^{-1}$. Calcium metal reacts with water to form calcium hydroxide according to $\mathrm{Ca}+2H_{2}O⟶{\mathrm{Ca}(\mathrm{OH})}_{2}+H_{2}$. To prevent any precipitation during the experiment, a water‐to‐solid ratio of 10,000 was maintained by dissolving 50mg of $C_{3}S$ in a volume of 500mL water. Samples of approximately 2.5mL each were collected at specified time intervals (see below) following the addition of the solid. After removal of the sample from the dissolution flask, the sample was immediately filtered through a syringe filter with a polyethersulfone (PES) membrane and a pore size of 0.2μm. The first sample was obtained 30 seconds after the solid was introduced, and subsequently, the sampling interval was extended to once per minute during the initial 10 minutes. Additional samples were collected at 12.5, 15, and 20 minutes after the initial contact between the solid and the solution. For ICP‐OES measurements, 1mL of each sample was mixed with 1mL of $6\;\mathrm{mol}\;L^{-1}$ nitric acid (${\mathrm{HNO}}_{3}$), diluted with 8mL of water and analyzed using an ICP‐OES 5800 (Agilent Technologies, Santa Clara, California, USA). Before sample measurement, the instrument was calibrated using a multipoint calibration procedure. Calibration solutions with concentrations of 50, 10, 1, and $0.1\;\mathrm{mg}\;L^{-1}$ were produced by dilution of $1000\;\mathrm{mg}\;L^{-1}$ single element standards for calcium (Calcium, plasma standard solution, Specpure, Thermo Fisher Scientific) and silicon (Silicon Standard for ICP, Sigma‐Aldrich) with ultrapure water and ${\mathrm{HNO}}_{3}$. Matrix effects were corrected using yttrium (Yttrium ICP standard, certipur, Merck) as an internal standard. The silicon concentration evolution over time is fitted using a simple first‐order equation^[^ ^28^ ^]^ of the form^[^ ^29^ ^]^ (Figure S9):

(6) $${\left[\mathrm{Si}\right]}_{t}={\left[\mathrm{Si}\right]}_{s}\left(1-e^{-kt}\right)$$

${\left[\mathrm{Si}\right]}_{t}$ (in $\mathrm{mol}\;L^{-1}$) is the silicon concentration measured at time t (in s), ${\left[\mathrm{Si}\right]}_{s}$ (in $\mathrm{mol}\;L^{-1}$) is the experimentally fitted equilibrium concentration (i.e., corresponding to the solubility of C‐S‐H, as determined by Kulik et al.^[^ ^30^ ^]^ (Figure S10), and k (in $s^{-1}$) is the rate constant of dissolution. The dissolution rate (r, in $\mathrm{mol}\;m^{-2}\;s^{-1}$) is then calculated from the fitted silicon concentration using Equation 7.^[^ ^31^ ^]^

(7) $$r=\frac{({\left[\mathrm{Si}\right]}_{t_{i}}-{\left[\mathrm{Si}\right]}_{t_{\left(i-1\right)}})\cdot V}{GSA\cdot \Delta t\cdot m_{C_{3}S}}\cdot \frac{1}{\nu }$$

where Δt is the time interval between $t_{i}$ and $t_{\left(i-1\right)}$ (in s), V the Volume of the reaction mix, $m_{C_{3}S}$ is the initial mass of $C_{3}S$ introduced in the reactor (in g), and GSA is the geometric surface area (in $\;m^{2}\;g^{-1}$) of the $C_{3}S$ sample estimated by dividing the specific surface area (SSA) measured by BET by a factor of 2.5,^[^ ^31^ ^]^ as proposed in a previous study.^[^ ^29^ ^]^ Note that all the dissolution rates displayed from hereon were determined at Ca and Si concentration range below the solubility limit of C‐S‐H as determined by Kulik^[^ ^30^ ^]^ and by Haas and Nonat^[^ ^32^ ^]^ (Figure S11).

### 2.3 Thermodynamic Modeling

Thermodynamic modeling of the dissolution systems was performed with PHREEQC^[^ ^33^ ^]^ using the CEMDATA18 database^[^ ^34^ ^]^ in order to calculate the ion activity product (Π) (Equation 8) during the $C_{3}S$ dissolution reaction (Equation 1).

(8) $$\Pi ={\left\{{\mathrm{Ca}}^{2+}\right\}}^{3}\cdot {\left\{{\mathrm{OH}}^{-}\right\}}^{6}\cdot \left\{H_{4}{\mathrm{SiO}}_{4}\right\}$$

where $\left\{{\mathrm{Ca}}^{2+}\right\}$, $\left\{{\mathrm{OH}}^{-}\right\}$, and $\left\{H_{4}{\mathrm{SiO}}_{4}\right\}$ are the ion activities in solution. To correctly model the polymer‐containing solutions, the calcium complexation by the negatively charged functional groups of the polymers must be considered. Thus, the calcium complexation as a function of the total calcium amount in solution was estimated for all polymer‐containing solutions using the results acquired during the CH nucleation experiments, as detailed in the next section. In the polymer‐containing systems, the measured calcium concentration deviates from the expected calcium concentration (Figure S5) at the beginning of the experiment (i.e., when less than $10\;m\mathrm{mol}\;L^{-1}$ of ${\mathrm{CaCl}}_{2}$ and $20\;m\mathrm{mol}\;L^{-1}$ of NaOH have been introduced in the solution). This deviation is fully attributed to calcium complexation. The amount of calcium complexed (in %) is therefore calculated using the following equation:

(9) $${\mathrm{Ca}}_{\mathrm{complexed}}=100\cdot \frac{{\left[\mathrm{Ca}\right]}_{\mathrm{added}}-{\left[\mathrm{Ca}\right]}_{\mathrm{measured}}}{{\left[\mathrm{Ca}\right]}_{\mathrm{added}}}$$

where ${\left[\mathrm{Ca}\right]}_{\mathrm{added}}$ is the amount of calcium introduced in the solution (in $m\mathrm{mol}\;L^{-1}$) and ${\left[\mathrm{Ca}\right]}_{\mathrm{measured}}$ is the calcium concentration (in $m\mathrm{mol}\;L^{-1}$) measured by the calcium electrode (as detailed in the next section). The resulting curves (Figures S6 and S7) are used to assess the calcium complexation as a function of the polymer type, the initial Ca(OH)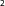 concentration, and the amount of $C_{3}S$ dissolved at the estimation of the dissolution rate. Note that, similarly to Marchon et al.,^[^ ^20^ ^]^ the potential complexation of silicon aqueous species by the polymers is neglected here. The contribution of the near‐neutral calcium‐polymer complexes to the ionic strength is also neglected.

### 2.4 Controlled Nucleation Experiments

To study the nucleation and crystal growth processes, a titration setup (OMNIS, Metrohm) equipped with two automatic burettes (Dosing unit, 20mL), pH (Unitrode), and a calcium ion selective electrode (Combined polymer membrane electrode) was used. An in‐house built double jacketed reaction vessel with a capacity of 250mL coupled to a thermostat (JULABO 200F) was used to maintain a constant solution temperature of $25.0\pm 0.1^{\circ }C$. The reaction vessel was closed with a lid with openings for the electrodes, and a gentle flow of dry nitrogen was applied to the surface of the solution to avoid carbonation during the experiment. The pH electrode was calibrated daily using three standard buffers of pH 4, 7, and 10 (Chemsolute, Th. Geyer). The calibration of the Ca ISE was repeated three times before each experiment by dosing ${\mathrm{CaCl}}_{2}$ solution ($0.5\;\mathrm{mol}\;L^{-1}$ for CH, $0.03\;\mathrm{mol}\;L^{-1}$ for C‐S‐H) into 100mL water (100mL water + 20mL NaOH ($1\;\mathrm{mol}\;L^{-1}$) for C‐S‐H). For C‐S‐H experiments, the calibration was performed at a similar (high) pH as the titration experiment. The calibration can, therefore, be done on a total Ca concentration basis, assuming that the calcium speciation variation and the activity coefficient variation between the calibration solution and the titration solution are negligible. These assumptions are supported by the good agreement between calcium measurement from the calcium probe and from ICP measurements (refer to Figure S14 for more details). For the CH experiment, the calibration is done in circumneutral conditions, while the titration occurs in basic conditions. Therefore, the calibration has to be done on an activity basis to ensure compatibility with the titration experiment. The output of the titration experiment is thus a ${\mathrm{Ca}}^{2+}$ activity curve. ${\mathrm{Ca}(\mathrm{OH})}^{+}$ activity is then calculated from ${\mathrm{Ca}}^{2+}$ and ${\mathrm{OH}}^{-}$ activities considering the stability constant

(10) $${\mathrm{Ca}}^{2+}+H_{2}O⟶\mathrm{Ca}{\left(\mathrm{OH}\right)}^{+}+H^{+}\;\mathrm{pK}=-12.78$$

The activity coefficients are then determined from the solution chemistry, allowing the ${\mathrm{Ca}}^{2+}$ and ${\mathrm{Ca}(\mathrm{OH})}^{+}$ concentrations to be summed to a total (free) calcium concentration. This calibration approach is supported by the good agreement between calcium measurement from the calcium probe and from ICP measurements (refer to Figure S15 for more details).

CH nucleation experiments were performed by a parallel addition of ${\mathrm{CaCl}}_{2}$ ($0.5\;\mathrm{mol}\;L^{-1}$) and NaOH ($1\;\mathrm{mol}\;L^{-1}$) into 100mL water at a constant speed of $0.4\;\mathrm{mL}\;\min^{-1}$. The solubility of Ca(OH)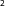 at the given conditions (pH, temperature, Ca activity) was calculated to $19.4\;m\mathrm{mol}\;L^{-1}$ using PHREEQC (V3) and the CEMDATA18 database.^[^ ^34^ ^]^ For the C‐S‐H nucleation experiments, a titrand solution of 100mL ${\mathrm{Na}}_{2}S{\mathrm{iO}}_{3}$ ($3.7\;m\mathrm{mol}\;L^{-1}$) and 20mL NaOH ($1\;\mathrm{mol}\;L^{-1}$) was used, and ${\mathrm{CaCl}}_{2}$ ($0.03\;\mathrm{mol}\;L^{-1}$) solution was dosed at a constant speed of $0.08\;\mathrm{mL}\;\min^{-1}$. For experiments with polymers, the corresponding amount of polymer solution was added to the titrand solution. The reproducibility of the measurements was checked for both reference systems (Figure S16). Picker et al. did similar nucleation experiments for C‐S‐H in the presence of different types of polymers (negatively charged polymers, positively charged polymers, and neutral polymers).^[^ ^22^ ^]^ Based on their observation for negatively charged polymers, the experimental deviations in the presence of additives is assumed to be in the same order, or slightly higher, than for the reference systems.^[^ ^22^ ^]^

**Figure 2:**
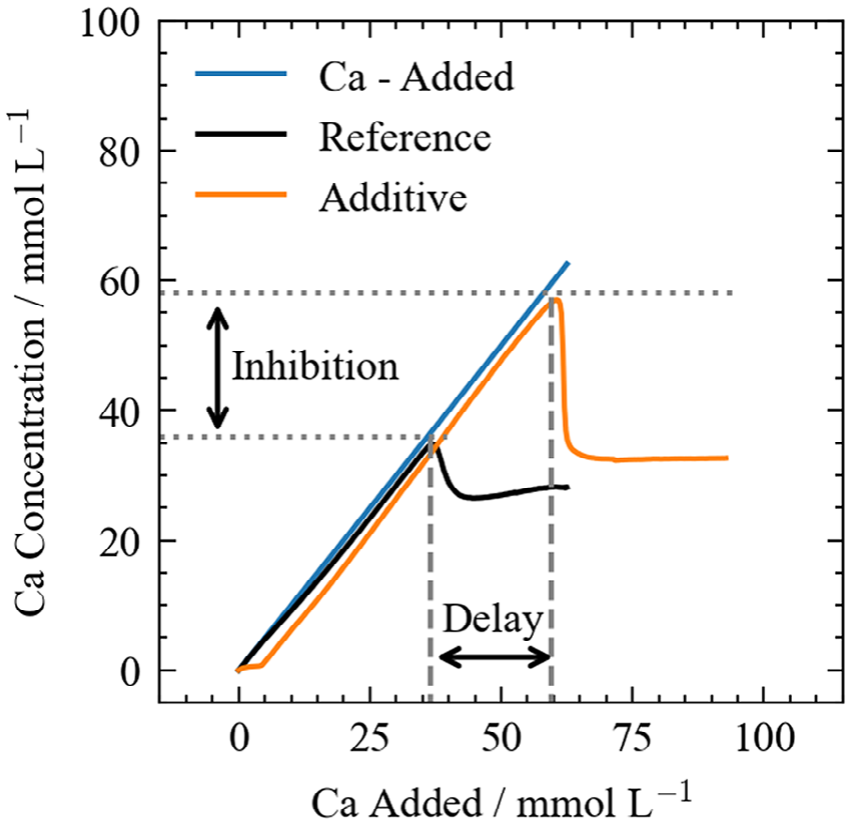
Scheme for portlandite nucleation in water and additive system, using the example of $4\;g\;L^{-1}$ PCE10. The inhibition can be quantified by the difference between measured calcium at the maxima and the delaying effect as the difference in added calcium at the maximum.

Several parameters may be extracted from the titration curves (Figure 2).^[^ ^29^ ^]^ First, the delaying factor (DF) is calculated from the excess calcium added at the nucleation onset using the following Equation 11:

(11) $$\text{DF}=\frac{{\left[{\mathrm{Ca}}^{2+}\right]}_{\max\;(\mathrm{added},\;\mathrm{additive})}}{{\left[{\mathrm{Ca}}^{2+}\right]}_{\max\;(\mathrm{added},\;\mathrm{ref})}}$$

A delaying factor greater than 1 indicates that the additive slows down the nucleation. Then, the inhibiting effect of the additives is investigated via the saturation index (SI) for CH nucleation only (Equation 12), where IAP is the ion activity product in the solution and K the solubility product of Ca(OH)
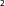

(12) $$\mathrm{SI}=\log\left(\frac{\mathrm{IAP}}{K}\right)$$

For the C‐S‐H nucleation experiments, the inhibiting effect is quantified via the inhibiting factor (IF), calculated from the calcium measured at the onset of nucleation (Equation 13). The saturation index cannot be calculated because we only measured the pH and calcium ion concentration continuously, while we did not measure the silicon concentration. Our previous work found that a discontinuous silicon measurement is insufficient to accurately calculate the saturation index vis‐a‐vis the C‐S‐H phase.^[^ ^29^ ^]^

(13) $$\text{IF}=\frac{{\left[{\mathrm{Ca}}^{2+}\right]}_{\max\;(\mathrm{measured},\;\mathrm{additive})}}{{\left[{\mathrm{Ca}}^{2+}\right]}_{\max\;(\mathrm{measured},\;\mathrm{ref})}}$$

An inhibiting factor greater than 1 indicates that the additive inhibits the nucleation.

### 2.5 Isothermal Heat‐Flow Calorimetry

Isothermal heat‐flow calorimetry measurements were performed following the procedure described by Nicoleau.^[^ ^35^ ^]^ 1.5g of ultrapure water was added to 3.0g of $C_{3}S$ (water to binder ratio, w/b  =  0.5) and manually mixed with a spatula for 30 seconds. About 1.5g of the paste was weighed into a plastic vial, sealed with a cap, and inserted into the calorimeter (8‐channel isothermal heat‐flow calorimeter, TAM Air, TA Instruments). The temperature was controlled at $20^{\circ }C$. The polymer solutions were diluted with water, and the dosages are indicated in wt.% per gram of $C_{3}S$.

### 2.6 Scanning Electron Microscopy of Portlandite Crystals

The titration experiments lead to the formation of turbid Ca(OH)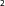 suspensions. The scanning electron microscopy (SEM) samples are prepared from this suspension directly after the completion of the titration experiment. The solid calcium hydroxide was isolated from the solution by centrifugation for 10 minutes at 8000 rpm (Biofuge primo R, Heraeus, Germany). The solid was washed two times using small amounts of isopropanol, dried under a constant flow of nitrogen overnight, and stored under a nitrogen atmosphere until further analysis. A small amount of the sample was mounted on a specimen holder for SEM analysis. The samples were sputter‐coated with gold, and the images were collected using a FESEM Zeiss Supra 50VP microscope using an electron acceleration voltage of 2kV.

### 2.7 Transmission Electron Microscopy of C‐S‐H

Transmission electron microscopy (TEM) was used to characterize the morphology of C‐S‐H precipitates. A 5μL aliquot of the suspension was removed during the titration experiments at the point of maximum Ca concentration, i.e., at the onset of nucleation (Figure S18). After removal from the titration solution, the sample was directly deposited on a carbon‐coated TEM grid (Carbon Film 300 Mesh, Copper, Sigma–Aldrich), and the sample was rapidly dried by immediately removing the supernatant solution by blotting with filter paper. The dried grids were stored in a closed box until measurement. The samples were analyzed within a week using a JEM 1400 plus microscope (JEOL) equipped with a ${\mathrm{LaB}}_{6}$ electron gun.

## 3 Results and Discussion

We first report on the polymer impact on the hydration kinetics of $C_{3}S$ characterized by isothermal heat flow calorimetry in Section 3.1. The next sections investigate the polymer impact on the dissolution of $C_{3}S$ (Section 3.2), followed by the influence on the nucleation of Ca(OH)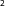 (Section 3.3) and C‐S‐H (Section 3.4).

### 3.1 Isothermal Heat Flow Calorimetry of $C_{3}S$

**Figure 3:**
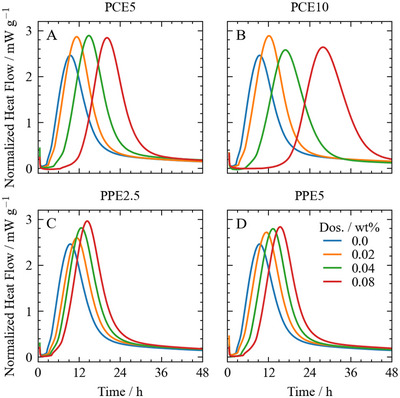
Heat flow curves for the reaction of $C_{3}S$ paste at a water to $C_{3}S$ ratio of 0.5. Different concentrations of various polymers were dissolved in the mixing water. The order of the polymers is (A) PCE5, (B) PCE10, (C) PPE2.5, and D) PPE5.

Isothermal heat flow calorimetry accurately determines the reaction kinetics of the hydration of $C_{3}S$. Superplasticizers in cement pastes are usually dosed in the range of 0.1%to0.2%‐bwoc (by weight of cement).^[^ ^36^ ^]^ However, in Portland cement, a significant amount of the polymer is consumed by, i.e., adsorbed on, nano‐ettringite, which forms in the first minutes as the reaction product of the tricalcium aluminate, $C_{3}A$, phase, and calcium sulfate.^[^ ^36^, ^37^, ^38^, ^39^ ^]^ Consequently, we set the polymer concentration from 0.02%to0.08% for the hydration experiments with pure $C_{3}S$. The obtained heat‐flow curves are shown in Figure 3.

A characteristic part of the $C_{3}S$ hydration reaction is the induction‐ or dormant period, a period of several hours with very low reactivity after the initial dissolution and the main silicate reaction. This dormant period can be seen in the first part of the heat‐flow curves, where almost no heat‐flow is observed within the first hours. The beginning of the acceleration phase coincides with the accelerated formation of portlandite and C‐S‐H.^[^ ^40^ ^]^

**Figure 4:**
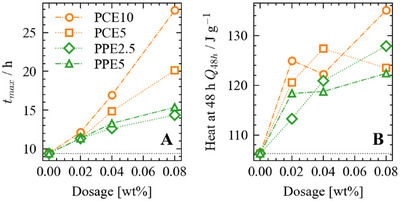
(A) Time points of the heat‐flow maxima ($t_{\max}$) and (B) the released heat after 48 hours per mass $C_{3}S$. $t_{\max}$ and the heat after 48 hours of the reference system (pure $C_{3}S$ paste without the presence of polymer) are shown as a grey dotted lines.

As the polymer dosage rises, the time required to reach the maximum heat flow $t_{max}$ increases (Figure 4A). This outcome reflects an extended retardation of the $C_{3}S$ reaction caused by the polymer. Also, note that the shape of the heat flow peaks remains similar for all experiments. The polymers with larger charge densities (PCE10 and PPE5) lead to a stronger increase than their lower charge counterparts. This observation is consistent with previously reported data for PCE interaction with $C_{3}S$.^[^ ^1^, ^3^, ^41^, ^42^, ^43^, ^44^, ^45^ ^]^ Additionally, the PCEs are stronger retarders than the phosphate polymers. The overall retarding trend at the highest dosage of 0.08% follows the order PCE10 > PCE5 > PPE5 > PPE2.5.

To quantify the impact of the different polymers on the duration of the hydration reaction, we determined both the time of the maximum heat flow ($t_{max}$) and the total heat after 48 hours (Figure 4). The heat after 48 hours increases for all polymers with increasing dosage from approximately $107\;J\;g^{-1}$ to a maximum of $135\;J\;g^{-1}$ for the highest dosage of PCE10. We note that these heat values are low compared to reported heat values for the hydration of $C_{3}S$. This is explained by the coarse particle size distribution $D_{50}=19.9\;\mu m$ of the $C_{3}S$ used here (the full characterization is found in the Supporting Information including the particle size distribution in Figure S3). A coarser $C_{3}S$ particle size distribution strongly reduces the early heat of hydration.^[^ ^46^ ^]^ As noted above, the heat after 48 hours in the presence of a polymer is higher than the heat of the reference system (Figure 4). This increase is equivalent to a large hydration degree and has been attributed to a templating mechanism of the polymer.^[^ ^45^ ^]^ Notably, at equivalent polymer dosages, the hydration degree at 48 hours does not show marked differences among the polymers

The observed retardation can be caused by the inhibition of the dissolution of $C_{3}S$, the inhibition of the nucleation of the hydrate phases (CH and C‐S‐H), or both mechanisms. The following sections individually explore the polymer impact on the $C_{3}S$ dissolution and nucleation of CH and C‐S‐H.

### 3.2 Dissolution of $C_{3}S$

**Figure 5:**
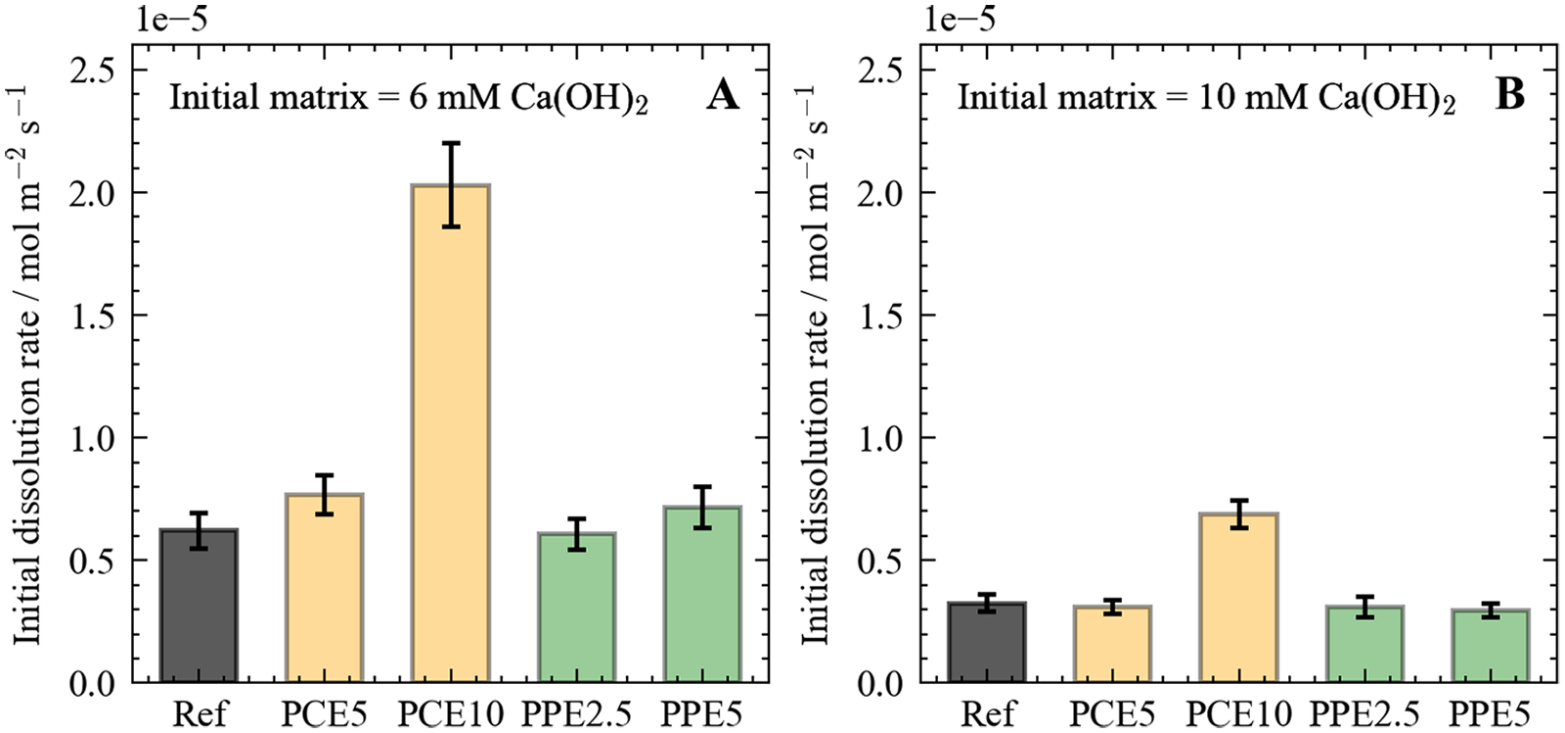
The dissolution of $C_{3}S$ under the influence of different superplasticizers in solutions with an initial Ca(OH)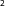 concentrations of $6\;m\mathrm{mol}\;L^{-1}$ (A) and $10\;m\mathrm{mol}\;L^{-1}$ (B). The polymer concentration was set to $4\;g\;L^{-1}$.

$C_{3}S$ dissolution was studied at highly diluted conditions (i.e., at a water‐to‐solid ratio of 10,000) to prevent C‐S‐H and CH formation. We first evaluated the polymer impact on the $C_{3}S$ dissolution rate in an aqueous solution containing $6\;m\mathrm{mol}\;L^{-1}$ Ca(OH)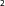 and a polymer concentration of $4\;g\;L^{-1}$ (Figure 5A). This concentration was selected based on analogous experiments reported in the literature^[^ ^20^ ^]^ and corresponds to an additive dosage of 0.2%‐bwoc in cement pastes with a water‐to‐cement ratio of 0.5, which lies within the conventional dosage range. PCE10 shows the strongest effect on the dissolution rate of $C_{3}S$, with a rate more than three times higher than the rate measured in the reference system devoid of polymer. PCE5 and PPE5 show a minor enhancing effect on the dissolution rate within the experimental error, while PPE2.5 shows no significant effect. When increasing the initial Ca(OH)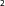 concentration to $10\;m\mathrm{mol}\;L^{-1}$, the impact of the polymers decreases even more (Figure 5B). PCE10 still induces an increase in the dissolution rate, which is more than two times higher than the reference rate. In contrast, no discernible effects are measured within errors for the other polymers, but at a higher PCE5 dosage of $12\;g\;L^{-1}$ (Figure S13), the rate recorded is two times higher than the reference rate.

In all polymer‐containing solutions, a significant fraction of the calcium initially present in the solution is complexed by the negatively charged functional groups of the polymers (Table S4). For a dosage of $4\;g\;L^{-1}$, PCE10 is the polymer that complexes the most calcium ions, while the other polymers show near‐similar calcium complexation capacity at a similar dosage (Table S4). That is because a polymer dosage of $4\;g\;L^{-1}$ results in a much higher monomer concentration in solution for PCE10 compared to the other polymers and, as a result, a much higher calcium complexation capacity (Figure S8). Calcium complexation effectively reduces the calcium activity in solution. The initial dissolution rate of $C_{3}S$ can be significantly impacted by the ${\mathrm{Ca}}^{2+}$ activity, as it affects the saturation state via the ion activity product (Π).^[^ ^21^, ^24^, ^47^ ^]^ In general, the dissolution rates measured in Ca(OH)
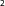
‐containing solutions follows a sigmoidal trend as a function of ln(Π) with an inflexion point at −69.3 (refer to Section S3.2 for more details).

**Figure 6:**
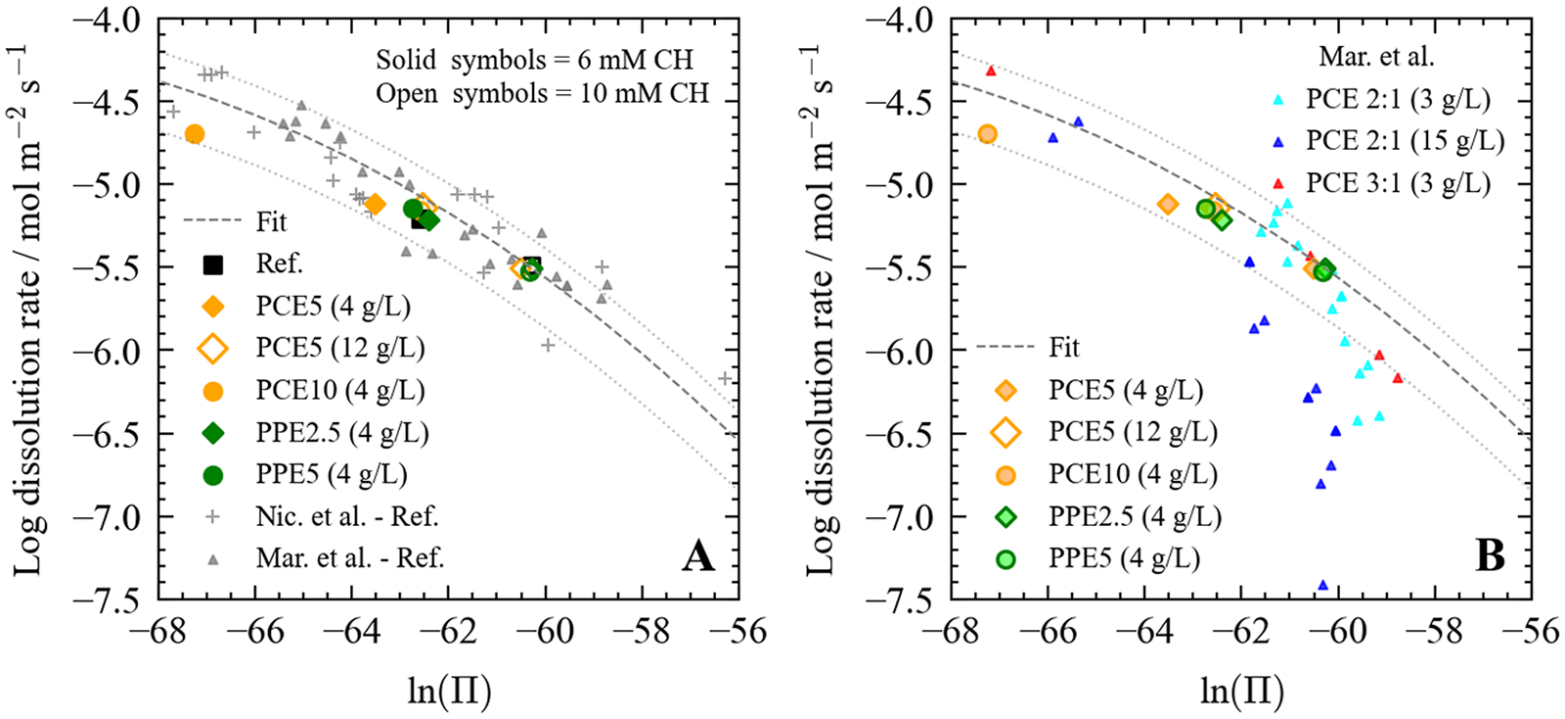
(A) The log of the initial dissolution rate as a function of the natural logarithm of the ion activity product (ln(Π)) in water or in the presence of polymer compared to reference datasets (i.e., Ca(OH)
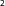
‐containing only solutions) from Nicoleau et al.^[^ ^24^ ^]^ and Marchon et al.^[^ ^20^ ^]^ (B) The log of the initial dissolution rate as a function of the natural logarithm of the ion activity product (ln(Π)) in the presence of polymer compared to different datasets from Marchon et al.^[^ ^20^ ^]^ The fitted dashed line was obtained using Equation S1. The dotted lines represent the 50% confidence interval. The rate values sourced from Marchon et al.^[^ ^20^ ^]^ were multiplied by a factor of 2.87 to account for the differences in experimental conditions (refer to Section S3.2 for more details).

All the experiments performed in our study were carried out in pore solution conditions above the threshold value of −69.3 (Table S4). Hence, any changes in calcium activity will strongly affect the dissolution rate of $C_{3}S$. To decouple potential additional effect(s) such as surface adsorption from the effect induced by the reduced calcium activity in solution, the dissolution rates are plotted as a function of ln(Π) in Figure 6 and compared to literature results.^[^ ^24^, ^47^ ^]^ In general, all the rates measured in the presence of a polymer align perfectly with our reference dataset and both literature datasets. In addition, for PCE5, the rates follow a similar trend as that of the reference within error, regardless of the polymer dosage. This suggests that (1) the variations observed in Figure 5 can be solely attributed to the calcium complexation capacity of the polymers, and (2) there is no significant additional impact of the polymers on the dissolution rate within the range of ln(Π) values studied herein. This is in contrast with the results of Marchon et al.,^[^ ^47^ ^]^ who observed a significant reduction in the dissolution rate of $C_{3}S$ in the presence of polymers, especially at higher dosages. A similar inhibiting effect was also observed with other retarding agents such as citrate and tartrate and ascribed to their adsorption at the surface of the $C_{3}S$.^[^ ^29^ ^]^ When comparing the results of Marchon et al.,^[^ ^47^ ^]^ with ours (Figure 6), it becomes apparent that this inhibiting effect occurs at a higher saturation state than that probed in the present study. Therefore, it seems that with large molecules such as PCEs and PPEs, the surface adsorption impacts on the dissolution rate of $C_{3}S$ needs to be investigated closer to saturation in future experiments.

### 3.3 Nucleation of Portlandite

#### 3.3.1 Titration Study of Polymer Influence on Portlandite Nucleation

The effect of the different polymers on the nucleation of Ca(OH)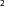 from a homogeneous solution was studied by creating supersaturated homogeneous solutions using an automated titration device.^[^ ^29^, ^48^ ^]^ As detailed in previous studies, a ${\mathrm{CaCl}}_{2}$ solution ($0.5\;\mathrm{mol}\;L^{-1}$) and a NaOH solution ($1\;\mathrm{mol}\;L^{-1}$) were introduced into a vessel containing 100mL of degassed ultra‐pure water at a delivery rate of $0.4\;\mathrm{mL}\;\min^{-1}$.^[^ ^29^, ^48^ ^]^ The polymers were added to the water at a concentration of $4\;g\;L^{-1}$.

**Figure 7:**
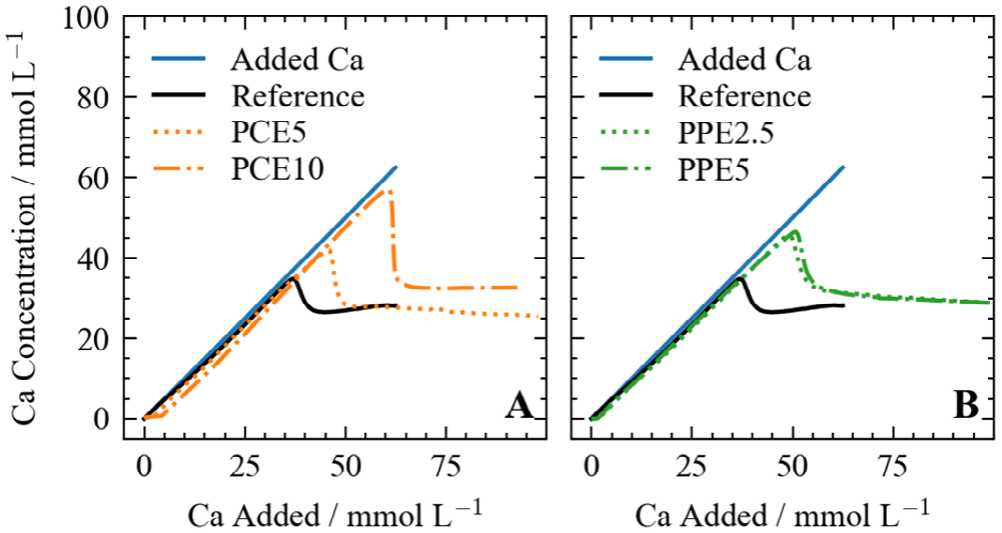
The nucleation of Ca(OH)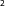 under the influence of (A) PCE5 and PCE10 (B) PPE2.5 and PPE5. All polymers were used at a concentration of $4\;g\;L^{-1}$.

The obtained nucleation curves are shown in Figure 7. Initially, the curves of the reference experiment without polymer (black line) and the theoretical curve (blue line) are congruent. With increasing amounts of calcium and hydroxide, the theoretical and experimental curves diverge, and the measured calcium concentration is slightly lower than the calculated calcium concentration. This could suggest a non‐classical crystallization pathway, as first reported for the crystallization of ${\mathrm{CaCO}}_{3}$ by Gebauer et al., where ions in solution form small, stable, solute precursor species, i.e., prenucleation clusters (PNCs), that later aggregate and form crystalline material.^[^ ^49^ ^]^ Madeja et al. have observed during their portlandite nucleation experiment an initial deviation in the bounded ${\mathrm{OH}}^{-}$ to bounded ${\mathrm{Ca}}^{2+}$ ratio that suggests the formation of ${\left[{\mathrm{Ca}(\mathrm{OH})}_{2+z}\right]}^{z-}$ species that could potentially include aqueous Ca(OH)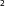 species, PNCs, and even aqueous species with z > 0.^[^ ^50^ ^]^ As the deviation shown in Figure 7 for the reference system is within the standard deviation of the measurement (Figure S16), we cannot formally conclude further on the formation of PNCs or any additional aqueous species in the reference system. The maximum of the titration curve (Figure 7) marks the onset of CH nucleation and, therefore, the precipitation of macroscopic solid particles. The crystal growth of the portlandite nuclei consumes calcium and hydroxide ions, leading to a significant decrease in free calcium concentration. A plateau is reached at the end of the experiment, corresponding to the solubility of the formed precipitate.

The complexation of calcium by the polymers is evident at the beginning of the experimental calcium curves, where the measured calcium concentration does not increase while the additives continue to complex calcium. The calcium concentration increases only when the maximum complexation capacity for each polymer is reached. A detailed view of the first part of the calcium curves is shown in Figure S5. As shown in Section 2.3, this deviation of the additive curves compared to the added calcium line is fully attributed to calcium complexation and no significant effects on PNCs could be observed in this early phase of the nucleation experiments.

Hindering effects due to additives can be quantified by the shift of the maximum, i.e., the nucleation onset, to higher calcium values along both the *x*‐axis (i.e., to higher values of added calcium, referred to as delaying factor in Table 2) and *y*‐axis (i.e., higher measurable calcium activity in solution before nucleation, quantified as ΔSI in Table 2) of Figure 7. The peak shift to higher added calcium concentrations indicates a delaying effect, while inhibiting effects manifest in a shift to higher free calcium activities (*y*‐axis). The delaying and inhibition effects of the polymers in this study are compiled in Table 2 (the detailed description of the method is given in Section 2.4). All the additives delay and inhibit the nucleation of portlandite compared to the reference, and both increased dosage and charge amount lead to larger delaying and inhibiting properties. The overall delaying effect follows the order PCE10 > PPE5 > PPE2.5 > PCE5, while the inhibiting effect follows the order PCE10 > PPE2.5 ≈ PPE5 ≈ PCE5. Similar nucleation studies for polyacrylic acid (PAA) have shown that a delaying factor of the same magnitude, i.e., 1.6, was reached by applying significantly smaller concentrations of $0.1\;g\;L^{-1}$ additive,^[^ ^50^ ^]^ or that (under different titration parameters) $0.01\;g\;L^{-1}$ of PAA can completely inhibit the nucleation of portlandite.^[^ ^51^ ^]^ It can be seen that PCE10 significantly increases the solubility of portlandite, indicated by the final calcium concentration in Table 2. Most likely, this increased solubility is a consequence of the complexation of calcium.^[^ ^52^ ^]^ The other additives show no significant influence on the solubility within the measurement error. An additional experiment with a polymer concentration of $12\;g\;L^{-1}$ was performed using PCE5 to estimate the influence of the polymer concentration (Figure S17). It can be seen that the delaying factor is only slightly higher. At the same time, the supersaturation is significantly increased, indicating a concentration‐dependent effect of the stabilization of the species in solution (Table 2).

**Table 2:** Portlandite nucleation experiments with different polymers.

Sample	Delaying Factor	SI	ΔSI	Final [Ca] $\left(m\mathrm{mol}\;L^{-1}\right)$
Reference	1.00	0.39	—	28.1
PCE5	1.24	0.60	+0.21	25.6
PCE5 ($12\;g\;L^{-1}$)	1.34	0.77	+0.38	25.6
PCE10	1.64	0.89	+0.51	32.7
PPE2.5	1.33	0.62	+0.24	28.0
PPE5	1.37	0.61	+0.22	28.2

Unless stated otherwise, a polymer dosage of $4\;g\;L^{-1}$ was applied. The delaying factor and the portlandite saturation indices (SI) were calculated at the maximum free calcium concentration (i.e., at the nucleation onset).

#### 3.3.2 SEM Micrographs of Portlandite

The connection between the influence of additives on the nucleation and the resulting morphology of the obtained crystals has been demonstrated in previous research.^[^ ^29^, ^53^ ^]^ To examine the morphology of the precipitates formed during the nucleation experiments, samples of the suspension were collected immediately after the titration experiments were stopped (end of the curves in Figure 7) and analyzed by SEM.

**Figure 8:**
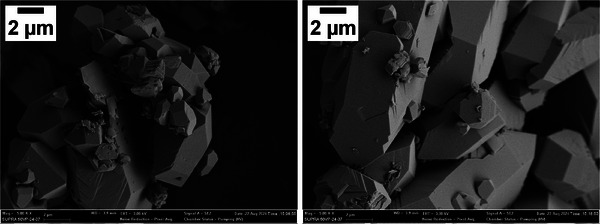
SEM images of portlandite from the reference system at 5000× magnification.

The portlandite crystals obtained from the reference system are shown in Figure 8. The observed morphology is consistent with the reported morphology of portlandite crystallized under similar conditions in the literature^[^ ^29^, ^51^, ^54^, ^55^ ^]^ and can be described as hexagonal prisms with irregular bipyramidal bases. The broad size distribution of the particles ranges from small particles of approximately 0.5×0.5×1 μm (height, width, length) to large particles with dimensions of 5×8×20 μm.

**Figure 9:**
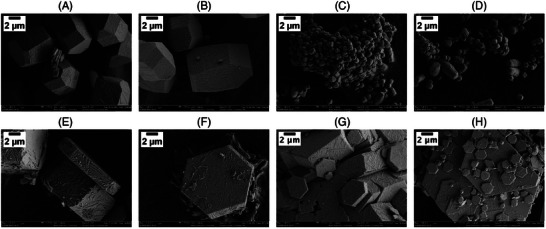
SEM images of Portlandite under the influence of different additives ($4\;g\;L^{-1}$) at 5000× magnification. (A), (B) PCE5 (C),(D) PCE10, (E), (F) PPE2.5 & (G), (H) PPE5.

The precipitates of the experiments under the influence of the different polymers are shown in Figure 9. At first glance, it is noticeable that the particle surfaces contain nanosized roughness features (see, for example, Figure 9E). As the separation of the particles was not done under inert conditions, it is possible that carbonation led to the formation of calcium carbonate on the surface of the particles.^[^ ^56^ ^]^ The particles precipitated in the presence of PCE5 (Figure 9A,B) show the same basic shape as the reference particles. In contrast to the reference, the size distribution is much more uniform with dimensions of approximately 5×8×10 μm for most of the particles. Increasing the charge of the polymer (i.e., PCE10 in Figure 9C,D) leads to a significant decrease of the particle size with diameter less than 1μm, while maintaining the shape of the individual crystals. Similar morphologies for portlandite crystallized under the influence of PCE superplasticizers were reported in the literature.^[^ ^57^ ^]^ The precipitates obtained from the nucleation experiments in the presence of PPEs show a significantly different shape in the form of thick hexagonal platelets. For PPE2.5 (Figure 9E,F), particles with a uniform diameter of approximately 12μm and varying thickness of 2to4 μm are formed. Similar to the trend in the PCEs series, increasing the charge leads to the precipitation of smaller particles (PPE5 in Figure 9G,H). In this case, two species are formed: thin, small hexagonal platelets and agglomerated structures of large hexagonal particles with a thickness of approximately 4μm. In summary, for all polymers, the formation of less intergrown particles indicates a stabilizing effect of the additives on the in situ formed nuclei. The growth of large isolated crystals in the presence of the lower‐charged polymers can be either attributed to the production of fewer nuclei or a less strong stabilization of small nuclei, resulting in an Ostwald's ripening to bigger particles. For higher charged molecules, either more (PPE) or solely (PCE) small individual particles were observed, indicating a strong stabilizing effect of the formed nuclei, even in the stage of crystal growth. Despite this stabilization, these particles formed significantly more agglomerated structures.

### 3.4 Nucleation of C‐S‐H

#### 3.4.1 Titration Study on C‐S‐H Nucleation with Polymers

**Figure 10:**
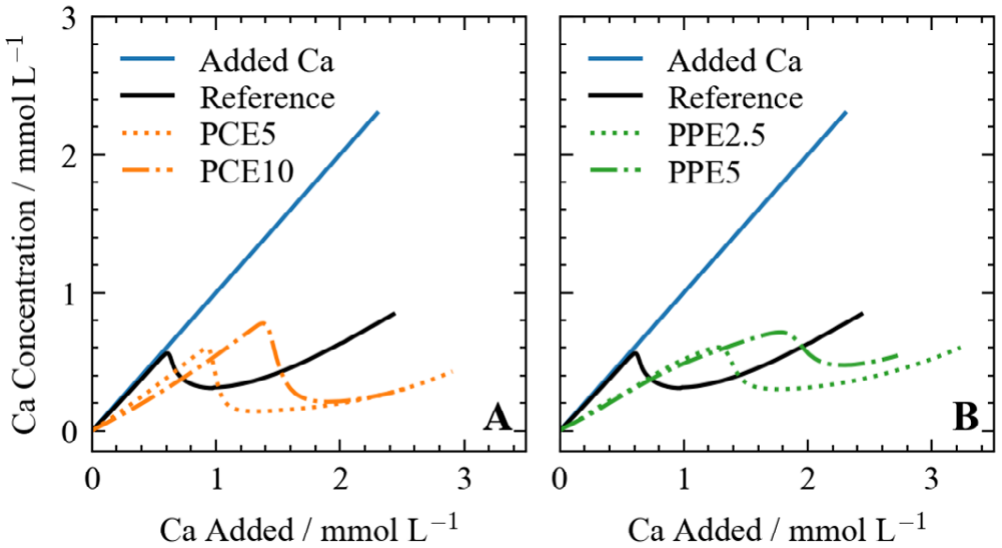
The nucleation of C‐S‐H under the influence of (A) PCE5 and PCE10 (B) PPE2.5 and PPE5. All polymers were used at a concentration of $4\;g\;L^{-1}$.

The nucleation of C‐S‐H from homogeneous solution was studied following an adapted procedure from Picker et al.^[^ ^22^ ^]^ The nucleation was achieved by slowly introducing a ${\mathrm{CaCl}}_{2}$ solution ($0.03\;\mathrm{mol}\;L^{-1}$) into a solution of 100mL of ${\mathrm{Na}}_{2}{\mathrm{SiO}}_{3}$ ($3.7\;m\mathrm{mol}\;L^{-1}$) and 20mL NaOH ($1\;\mathrm{mol}\;L^{-1}$) at a dosing rate of $0.08\;\mathrm{mL}\;\min^{-1}$. For the reference, a linear increase of the free calcium concentration up to a maximum, marking the onset of C‐S‐H nucleation, is observed (black curve in Figure 10). The experimental curve does not deviate from the theoretical curve, suggesting the absence of prenucleation clusters. A non‐classical nucleation pathway was reported for C‐S‐H nucleation.^[^ ^22^, ^58^, ^59^, ^60^ ^]^ In our setup, at the highly alkaline conditions of pH 13, only a very small amount of PNCs is expected due to the electrostatic repulsion of the silicate monomers.^[^ ^22^ ^]^ In contrast to the portlandite curves, no plateau is reached at the end of the experiment. The fixed amount of silicate and hydroxide ions explains this. The calcium concentration starts to rise again once the solubility of C‐S‐H under the given conditions of silicate and hydroxide is reached.^[^ ^29^ ^]^

The slope of the curves in the presence of the polymers is significantly smaller than for the reference. As the PNC formation is very low under the given conditions, the main effect causing this decrease in the slope is assumed to be attributed to the calcium‐binding affinity of the molecules, which was already shown for other polymers studied by Picker et al.,^[^ ^22^ ^]^ and also observed for tartaric and citric acid.^[^ ^29^ ^]^ We determined the delaying and inhibiting factor for the different polymers (Table 3). For both PCEs and PPEs, an increase in charge leads to an increase in delaying and inhibiting the nucleation of C‐S‐H. The overall delaying effect follows the order PPE5 > PCE10 > PPE2.5 > PCE5, while the inhibiting effect follows the order PCE10 > PPE5 > PPE2.5 ≈ PCE5. Interestingly, all the polymers except PPE2.5 significantly affect the calcium concentration measured after nucleation. In the presence of the PCEs, the minimum concentration is significantly lower than the minimum concentration measured in the reference system. In contrast, in the presence of PPE5, the minimum concentration is higher than the reference. In the absence of silicon concentration measurement, it is difficult to say if this denotes a change in the C‐S‐H composition (i.e., Ca/Si ratio) and/or a change in solubility (i.e., amount of C‐S‐H precipitated). In contrast to the results for the portlandite nucleation, the increase in PCE5 concentration to $12\;g\;L^{-1}$ strongly affect the C‐S‐H nucleation curve Figure S17). The delaying factor is increased, while a significant decrease in the slope and the inhibition factor is found (Table 3), most likely due to the increased calcium complexation with increased polymer concentration.

**Table 3:** C‐S‐H nucleation experiments with different polymers.

	Delaying factor	Inhibiting factor	Slope	Calcium conc. at minimum $\left(m\mathrm{mol}\;L^{-1}\right)$
Reference	1.00	1.00	0.95	0.31
PCE5	1.55	1.07	0.64	0.14
PCE5 ($12\;g\;L^{-1}$)	1.87	0.69	0.34	0.14
PCE10	2.30	1.39	0.51	0.21
PPE2.5	2.13	1.07	0.54	0.30
PPE5	2.95	1.27	0.52	0.48

Unless stated otherwise, a polymer dosage of $4\;g\;L^{-1}$ was applied. The delaying and inhibiting factors were calculated at the maximum free calcium concentration (i.e., at the nucleation onset). The slopes were calculated for the linear part of the calcium curves prior to the nucleation onset.

#### 3.4.2 TEM Images of C‐S‐H

**Figure 11:**
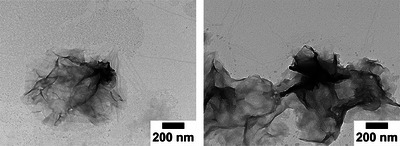
TEM images of the C‐S‐H reference at 30,000 × magnification.

In contrast to portlandite crystals, C‐S‐H precipitated from homogeneous solution form particles with a few nanometers in size,^[^ ^59^, ^61^ ^]^ making it impossible to analyze their morphology via SEM. TEM was used instead to analyze the morphology of the obtained C‐S‐H nanoparticles. At the local maximum of each nucleation experiment shown in Figure 10, i.e., the onset of nucleation, samples were taken from the reaction mix for TEM analysis. A detailed description of the sample preparation is shown in Section 2.7. The characteristic sheet‐like structure of C‐S‐H^[^ ^59^, ^62^, ^63^ ^]^ is shown for the reference sample in Figure 11.

**Figure 12:**
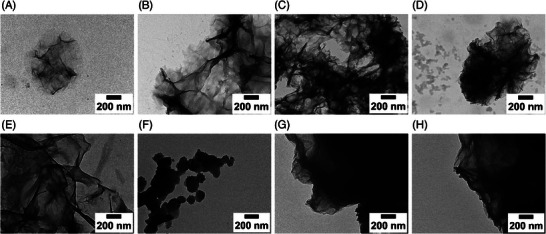
TEM images of C‐S‐H under the influence of different additives ($4\;g\;L^{-1}$) at 30,000 × magnification (A), (B) PCE5 (C), (D) PCE10, (E), (F) PPE2.5 & (G), (H) PPE5.

The C‐S‐H samples recovered from the polymer‐containing experiments are shown in Figure 12. In contrast to the reference, none of the samples shows these widespread unfolded sheets of C‐S‐H. Instead round agglomerates of small, wrinkled sheets are formed.

The C‐S‐H agglomerates of the less charged PCE5 (Figure 12A,B) are, with a diameter of approximately 400nm, double the size of the particles with PCE10 (Figure 12C,D). The morphology of the particles precipitated under the influence of the two different PPE polymers is more agglomerated. Increasing the charge of the polymer (PPE5 in Figure 12G,H) leads to a more agglomerated structure of C‐S‐H sheets. In summary, the formation of individual clustered particles for most of the polymers indicates their stabilizing effect on the crystal nuclei and the following crystal growth. Compared to the portlandite samples, a similar trend with a decrease in particle size could be observed for increasing the charge of the PCE samples.

## 4 Discussion

Superplasticizers not only have a dispersing and water‐reducing effect but also have a retarding effect on the reaction kinetics of the cement hydration.^[^ ^18^ ^]^ Here, all four phosphate‐ and carboxylate‐based superplasticizers (PPE and PCE) were indeed shown to retard the overall hydration of $C_{3}S$. Previous studies reported an inhibiting effect of PCEs on the dissolution rate of $C_{3}S$ ^[^ ^18^, ^20^ ^]^ that could explain their overall retarding effect. Upon first inspection, we observe an accelerating effect of the additives on the $C_{3}S$ dissolution (Figure 5), yet this acceleration is fully attributed to the calcium complexation by the polymers under alkaline conditions, which reduces the calcium activity in solution and the resulting degree of undersaturation. The dissolution rates measured in the presence of the polymers, when plotted against ln(Π), align perfectly with the dissolution rates measured in the reference system (Figure 6). Although our results would suggest at first glance that the polymers do not inhibit the dissolution rate, the combination of our results with literature data^[^ ^20^, ^24^ ^]^ suggests instead that any hindering effects of the polymers on $C_{3}S$ dissolution occur very close to saturation. This has to be further confirmed and quantified in future studies, particularly for PPEs that are not yet well studied to assess the contribution of the $C_{3}S$ dissolution inhibition to the retarding effect.

In contrast to the absent inhibiting polymer effect on the dissolution rate, we found a significant delaying and inhibiting influence of the polymers on the nucleation of portlandite and C‐S‐H. The polymers with the higher charge density induce stronger hindering effects to the nucleation, which corresponds to the trend found for the $C_{3}S$ hydration experiment.^[^ ^1^, ^3^, ^41^, ^42^, ^43^ ^]^ It has been proposed that, for portlandite, this hindering effect could be caused by the stabilization of PNCs.^[^ ^51^ ^]^ However, we believe that the formation of PNCs remains to be formally demonstrated in portlandite nucleation experiments. Madeja et al. have revealed the formation of ${\left[{\mathrm{Ca}(\mathrm{OH})}_{2+z}\right]}^{z-}$ species prior to the nucleation of portlandite that could encompass aqueous Ca(OH)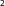 species, PNCs, and aqueous species with z > 0,^[^ ^50^ ^]^ yet they state that more work is necessary to fully elucidate the nature of solute species in the Ca(OH)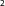/$H_{2}O$ system. Similarly, we cannot formally exclude nor validate the formation of PNCs in our portlandite nucleation experiments, yet the amounts, if formed, are expected to be low, with no significant stabilizing or destabilizing effects in the presence of the polymers. It has been suggested that this hindering effect could also be caused by the stabilization of amorphous precursors through the coordination of additives to calcium ions on the surface of the precursor particles. This hypothesis stems from experimental and atomistic modeling studies of the nucleation of calcium hydroxide,^[^ ^51^ ^]^ calcium carbonate,^[^ ^64^ ^]^ and calcium oxalate.^[^ ^65^ ^]^ Nicoleau et al. have proposed a sensibly similar hypothesis to explain the retarding effect of polymers (e.g., poly(acrylic acid), poly(acrylamide), and poly(aspartic acid)) on the nucleation of gypsum.^[^ ^66^ ^]^ They suggested that the retarding effect was most likely caused by the interaction of the polymer molecules with the different nucleating species in solution, therefore hindering their aggregation into the crystal nuclei required for crystallization.^[^ ^66^ ^]^ This retarding effect of negatively charged polymers is consistent with our portlandite and C‐S‐H nucleation results. It also aligns with the findings of Picker et al., who studied the impact of a range of negatively charged polymers (e.g., poly(acrylic acid), poly(acrylamide‐co‐acrylic acid), etc.) on C‐S‐H nucleation.^[^ ^22^ ^]^ We can therefore hypothesize that negatively‐charged polymers also interact with portlandite and C‐S‐H nucleating species in solution, which hinders the nucleation. This hypothesis is supported by the formation of small, individual particles of portlandite and C‐S‐H in the presence of PCE10 (the polymer with the highest charge density, Figures 9C,D and 12C,D).

Although the experimental differences between carboxylate and phosphate superplasticizers are noticeable, we do not observe fundamental differences concerning their effects. The most pronounced differences between the carboxylate and phosphate polymers are found in the C‐S‐H nucleation data. Here, the phosphate polymers show a significantly higher delaying factor than their respective carboxylate counterparts (the inhibiting factor is similar). It must be noted that the phosphate polymers deviate more strongly from the intended chemical composition due to the impurity of the phosphate monomer. Therefore, although the polymers were intended to have the same charge‐to‐mass ratio at full deprotonation of the functional groups, PPE5 has a significantly lower charge than PCE10 due to the monomer quality. Finally, none of the delaying and inhibiting trends observed for portlandite and C‐S‐H nucleation align with the overall retarding trend observed for $C_{3}S$ hydration (PCE10 > PCE5 > PPE5 > PPE2.5). Therefore, we can conclude that the titration experiments demonstrate the polymer‐induced inhibiting effect and also capture the stronger effect of the higher charge density polymers. However, because the titration experiments do not reproduce the correct polymer order found for the retardation of the $C_{3}S$ hydration, they are not sufficient to elaborate the relationship between the polymer structure and its impact on the $C_{3}S$ hydration. Additional experiments are needed to elucidate the effect of polymer structure parameters on the nucleation of hydrate phases and the consequences for the retardation of cement hydration.

Finally, morphology studies of portlandite and C‐S‐H using electron microscopy have shown significant effects of the polymers on the morphology of the obtained particles. Increasing charge density generally leads to decreased portlandite particle size, similar to previously reported data for calcium hydroxide and PCE.^[^ ^57^ ^]^ At the highest charge density, i.e., for PCE10, agglomerated, nano‐sized portlandite crystals were observed. For C‐S‐H, the particle shape changes from extended, wrinkled nano‐sheets without polymer to round, compact agglomerates in the presence of the superplasticizers. Higher charge density led to smaller agglomerates both for PCE and PPE.

## 5 Conclusion

Superplasticizers show a retarding effect on cement hydration and the hydration of pure clinker phases such as $C_{3}S$. All four phosphate‐ and carboxylate‐based superplasticizers (PPE and PCE) in our study showed a retarding effect on $C_{3}S$ hydration. In particular, we showed that (1) both PPEs and PCEs inhibit the nucleation of portlandite and C‐S‐H, and (2) a larger polymer charge density increases the nucleation inhibition for both hydrate phases. This pattern fits to the observed structure–activity relationship for the retardation of $C_{3}S$. The morphologies of the precipitates confirm the strong polymer influence on nucleation and crystal growth. We find smaller primary structures with increasing charge density of the polymer. For portlandite, the particle size decreases from approximately 20μm for the basal hexagonal face to a few hundred nanometers for the PCE with the largest charge density. Similarly, the highest‐charge polymer leads to spherical C‐S‐H particle agglomerates with approx. 200nm in diameter, while the C‐S‐H sheets of the reference sample reach a lateral size of a micrometer. Finally, and in contrast to previous studies, we do not find a hindering polymer effect on the dissolution of $C_{3}S$. We note, however, that the limited experimental parameter space of our study can tentatively explain this observation. Consequently, our results do not disprove the previous observation that superplasticizers can inhibit the $C_{3}S$ dissolution close to saturation but highlight the critical importance of these experimental parameters. Future studies are needed to extend the experimental evidence for dissolution experiments at high polymer dosages and saturation conditions close to the solubility of the C‐S‐H phase.

## Conflict of Interests

The authors declare that there are no conflicts of interest.

## Supporting information

Supporting Information

## Data Availability

The data that support the findings of this study are available from the corresponding author upon reasonable request.
