# Supplementary material for: Influence of Polymeric Dispersants on the Dissolution Rate of Tricalcium Silicate and the Nucleation of Calcium‐Silicate‐Hydrate and Portlandite
Source: Chemistry. 2025 Apr 25;31(27):e202500207. doi: 10.1002/chem.202500207 (PMC12080329; doi:10.1002/chem.202500207)
Supplement: Supplementary file 1 — Supporting Information [file CHEM-31-e202500207-s001.pdf]

Supplementary Information: Influence of Polymeric Dispersants on the Dissolution Rate of Tricalcium Silicate and the Nucleation of Calcium-Silicate-Hydrate and Portlandite

Andreas Vohburger[1], Marie Collin[1], Olivia Rindle[1] and Torben Gädt[1,*]

[1] Chair for the Chemistry of Construction Materials, TUM School of Natural Sciences, Technical University of Munich, Lichtenbergstraße 4, 85748 Garching, Germany

[*] Contact: torben.gaedt@tum.de

S1 Methods

S1.1 Purification of the Macromonomer

To remove methacrylic acid from the macromonomer, an ion exchanger was prepared by swelling it in water within a column. The column was then treated with a 5% NaOH solution until the outcome reached a pH of 14. Subsequently, the column was rinsed with water until the outcome reached a pH of 7. The MPEG5000-MA was then passed through the column, and the purified product was collected. The effectiveness of the purification process was confirmed using ^{1}H-NMR spectroscopy.

S1.2 Nuclear Magnetic Resonance (NMR) Spectroscopy

NMR spectra were recorded on a Bruker Advance III AV400US (400 MHz). Chemical shifts are reported in ppm (parts per million) relative to tetramethylsilane (for ^{1}H) and were referenced to the residual solvent peak as an internal standard.

S1.3 Monomer Synthesis

The phosphate monomer 2-(methacryloyloxy) ethyl phosphate (for simplicity, the reaction product is referred to as monohema phosphate, MHP, in the following) is prepared according to [1, 2] by placing

48.65 mL 2-hydroxyethyl methacrylate (HEMA) (52.05 g, 400 mmol) in a three-neck flask, followed by cooling to 0 °C. Afterward, 20.29 mL (41.79 g) polyphosphoric acid was placed in a dropping funnel and heated until the viscosity was low enough to be added dropwise within 10 min. After the addition of polyphosphoric acid was complete, the reaction mixture was kept at 0 °C for another 2 h. Subsequently, the viscous MHP sample was stored at 4 °C and used without further purification for the polyphosphate synthesis. The resulting product contains different side products which form during synthesis. The main side products that are formed by the reaction of HEMA with polyphosphoric acid are shown in Figure S1). Next to these products, polyphosphoric acid can form phosphoric acid (PA) or pyrophosphoric acid (PyPA). The composition of these products can be quantified using ^{31}P-NMR spectroscopy. The relative amounts of the different species can be determined by identifying the chemical shifts corresponding to each product and integrating the respective peaks. Given that ^{31}P has a natural abundance of 100 %, the NMR analysis benefits from the direct proportionality between the area under an NMR peak and the number of nuclei contributing to that resonance. The composition of the synthesized monomer is shown in Table S1.

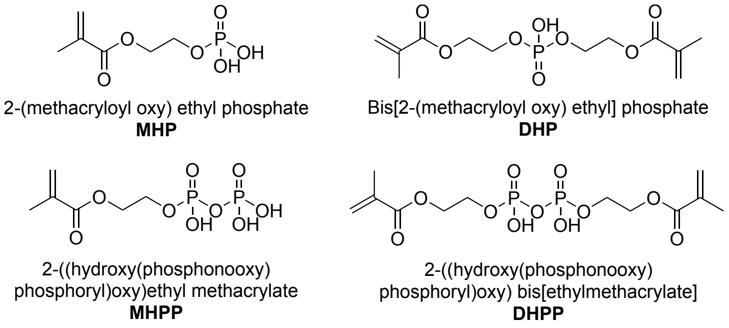

Figure S1: Chemical structure of the main polymerization active products of the phosphate-based monomer mixture.

Table S1: NMR signals for the phosphate-based monomer mixture (Figure S2) with their corresponding chemical shift and their amount in mol%. Due to the overlap of the peaks of the different pyrophosphoric species, a precise differentiation between these species is not possible. Consequently, their chemical shift and amount are grouped together.

Substance	Chemical shift [ppm]	Amount [mol%]
PA	0.0	29
Unknown	−0.1	3
MHP	−0.3	45
DHP	−0.4	7
THP	−0.5	3
Pyrophosphoric species	−11.4 - −11.5 and −11.9 - −12.0	13

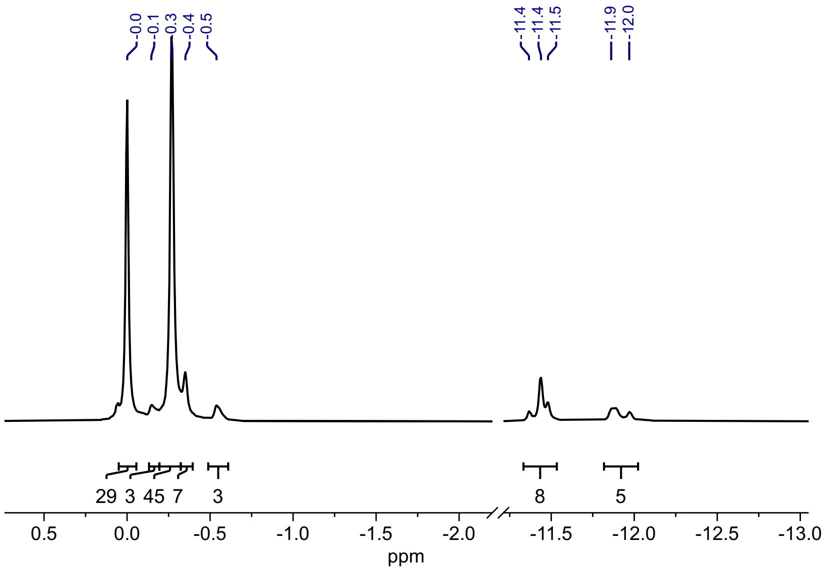

Figure S2: ^{31}P-NMR spectrum (D_2O) of the phosphate-based monomer.

S1.4 Polymer Synthesis and Characterization

The polycarboxylate and polyphosphate dispersants were synthesized according to [1, 2] by placing the respective amounts of charged monomer (methacrylic acid or monohema phosphate) and aqueous solution of Methoxy-poly(ethylene glycol)-methacylate (MPEG5000-MA, $M_n = 5000\,\text{g}\,\text{mol}^{-1}$, purified) in a round-bottom flask. The solid content of the solution is adjusted to 20 % by adding deionized water. The solution is heated to 80 °C and then 3-MPA and NaPS were added to start the polymerization. The reaction mixture was stirred for 1 h at 80 °C. After the polymerization, the solution was cooled to room temperature, and the pH value was adjusted to pH 7 with an aqueous 40 % sodium hydroxide solution.

For the purification of the polymers, tangential flow filtration (Vivaflow 50) with a molecular weight cut off of 10 000 Da was conducted for 8 h until the amount of unreacted monomer and macromonomer left in the retentate was negligible.

Table S2: Amount of chemicals which were used in the polymerizations. Based on the total amount of double bonds in the system, 2 % of NaPS as initiator and 6 % of 3-MPA as chain transfer agent were used.

Polymer	Charged Monomer (C) / mol	Macromonomer (E) / mol	Equivalence Ratio (C/E)
PCE5	MA 0.05	0.01	5:1
PCE10	MA 0.39	0.0039	10:1
PPE2.5	MHP 0.02	0.008	2.5:1
PPE5	MHP 0.06	0.012	5:1

The average monomer to co-polymer (C/E) ratio was determined by pH titration from pH 3.0 to pH 11.0 of an acidified polymer-containing solution with a NaOH-containing solution. Polycarboxylate do

not have a single pKa value, but rather multiple pKa ranging from 5.1 up to 7.8 depending on the chain length and the degree of protonation of the polymer [3]. For polyphosphate with 2 charges (e.g., MHP), the first pKas are very acidic (i.e., below pH 1.5) [4] and are not measured in the pH range probed here, but the second pKa values typically range from 5.5 to 8.4 [4]. The C/E ratio for polycarboxylate and polyphosphate is consequently equivalent to the molar amount of NaOH necessary to neutralize the polymer divided by the molar amount of polymer present in the solution.

The polymer weight (M_n, M_w) and PDI was analyzed with size exclusion chromatography on an SECcurity[2] GPC-System from PSS Polymer Standards Service GmbH. The system consists of an Agilent 1260 Infinity II Isocratic Pump equipped with an upstream degasser, an Agilent 1260 Infinity II Vialsampler, a tempered column compartment at 35 °C containing a SUPREMA pre-column (8×50 mm; particle size: 5 µm) and three main columns (8×300 mm; particle size: 5 µm; pore sizes: 30 Å, 1000 Å, 1000 Å). An Agilent 1260 Infinity II refraction index detector and a PSS SLD7100 multi-angle static light scattering detector were used for detection. All measuring angles (35°, 50°, 75°, 90°, 105°, 130°, and 145°) are used for the calculation of the molecular weights from the light scattering detector. The eluent is an aqueous buffer solution of 0.07 mol L^{-1} Na_2HPO_4 and 200 mg L^{-1} NaN_3. A flow rate of 1 mL min^{-1} was applied. The system is calibrated by classic calibration with ReadyCal PEG standards. The light scattering detector was calibrated with a pullulan standard (molecular weight = 110 000 g mol^{-1}). The reference standards were obtained from PSS Polymer Standards Service GmbH.

S1.5 Synthesis of Tricalcium Silicate

The synthesis of tricalcium silicate (C_3S) was conducted according to a modified synthesis protocol of Li et al. [5]. A detailed description of the synthesis procedure is given elsewhere [6, 7]. Analytical grade $CaCO_3$ and fused silica (Amosil 510, HPF Minerals) were mixed at a molar ratio of Ca/Si = 3. The solids were mixed with water (water to solid ratio, w/s = 2) in a ball mill (AAM-WA 350, AAM Mahltechnik, Germany, 24 h, 70 rpm, 12.5 mm ZrO_2 balls) and the obtained paste was filled into four cylindrical silicone molds, left to dry for two days, unmolded, and dried to constant weight at 120 °C. The cylinders were transferred to platinum crucibles and sintered at 1550 °C for 10 h. The reaction product of the solid-state reaction was quenched at 1500 °C using pressurized air. The cooled discs were crushed in a jaw crusher and finely powdered using a ball mill (90 min, 70 rpm, 12.5 mm ZrO_2 balls). The powder was sieved through a mesh size of 90 µm and stored in a Schlenk tube under inert conditions. The phase composition of the final product was confirmed using powder X-ray diffraction. Rietveld analysis reveals a composition of 97.2 wt.% of C_3S (triclinic), 2.4 wt.% of β-C_2S, and less than 0.5 wt.% CaO. The BET specific surface area (SSA) was determined to 0.083 m^2 g^{-1} using a NovaTouch LX4 surface area and pore size analyser (Quantachrome Instruments). The particle size distribution was determined by laser diffraction analysis in isopropanole (CILAS 1064 Lasergranulometer) (Figure S3). We find the following values: D_{10} = 2.94, D_{50} = 19.87, D_{90} = 38.79 µm.

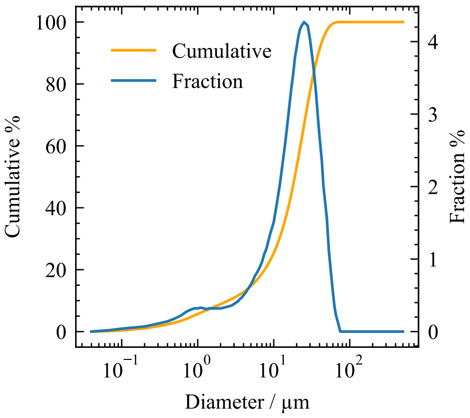

Figure S3: Particle size distribution of C_3S powder.

S2 Calorimetry

All polymers increase the early heat until 48 h of the C_3S hydration. The strongest effect is found for PCE10 at the largest dosage. The effect is tentatively ascribed to a templating effect of the polymer [8]. All polymers are added with the mixing water, i.e., in the so-called direct addition mode. It is conceivable that the presence of non-adsorbed polymer leads to partial nucleation of hydrate phases in the pore solution, stabilized by the PCE.

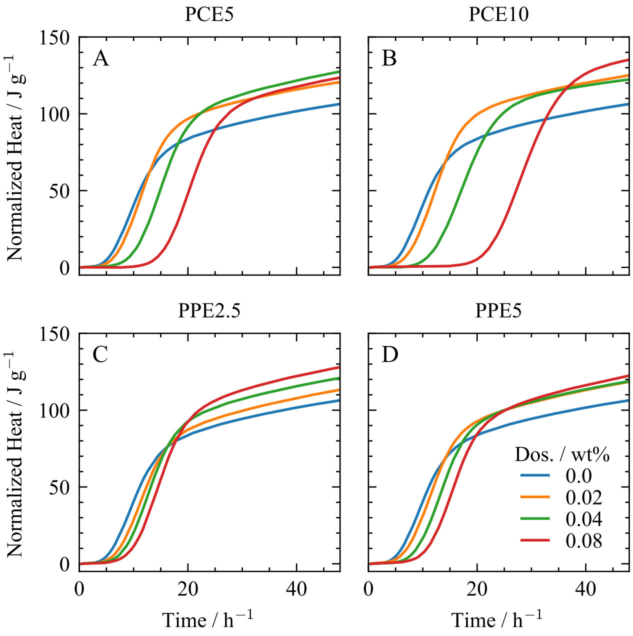

Figure S4: Isothermal heat flow calorimetry. The heat at 40 min was arbitrarily set to zero to remove the thermal effects from the initial sample insertion.

S3 Dissolution

S3.1 Calcium complexation determination

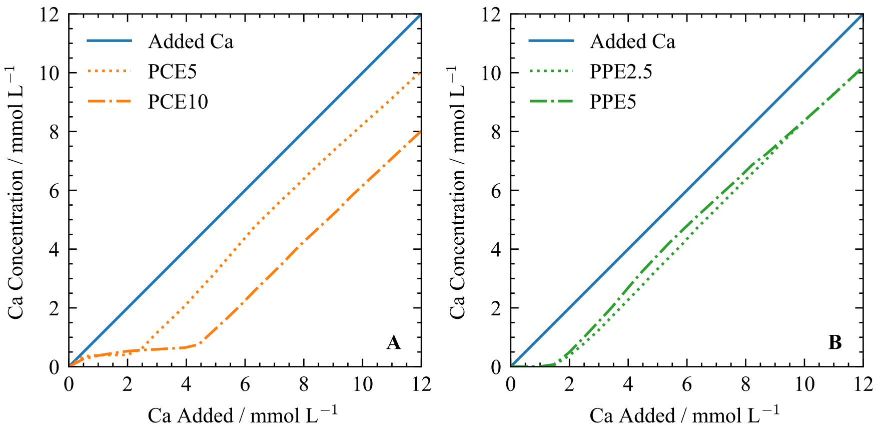

Figure S5: Zoom in on the measured calcium concentration in the presence of (A) two different PCEs with C/E = 5:1 and C/E = 10:1, and (B) two different PPEs with C/E = 2.5:1 and C/E = 5:1 used at a concentration of $4\,g\,L^{-1}$ in the portlandite nucleation experiments.

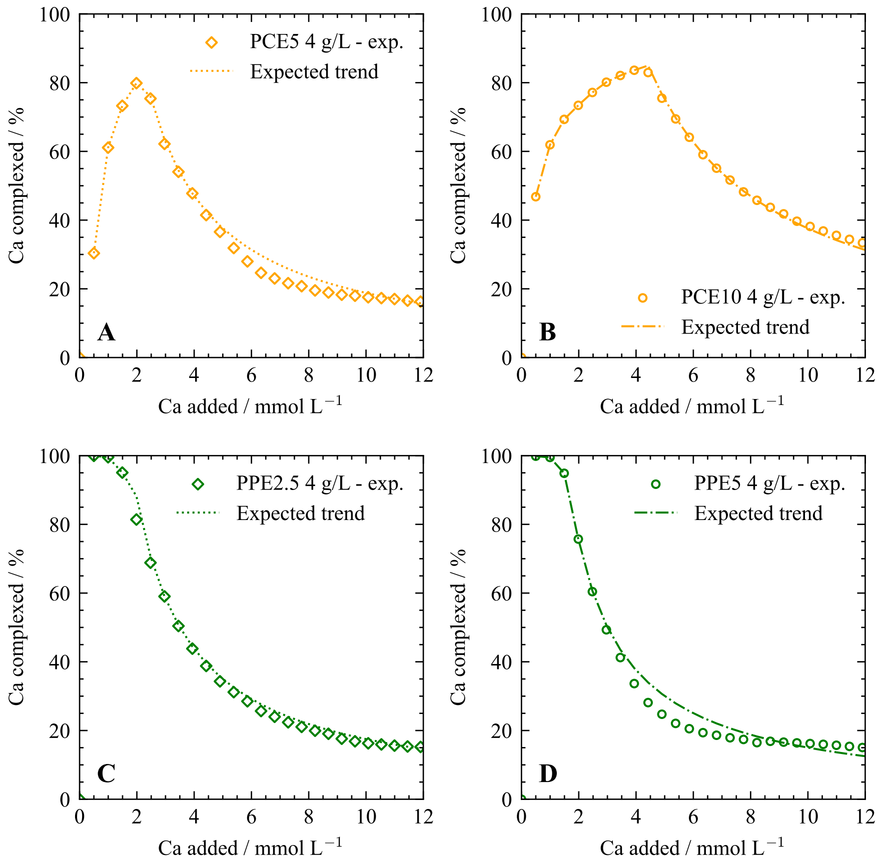

Figure S6: The percentage of calcium complexed as a function of calcium added in the portlandite nucleation experiments in solutions containing 4 g L^{-1} of (A) PCE5, (B) PCE10, (C) PPE2.5, or (D) PPE5. The symbols are experimental measurements, while the lines are the expected trend from the maximal calcium capacity of the polymers.

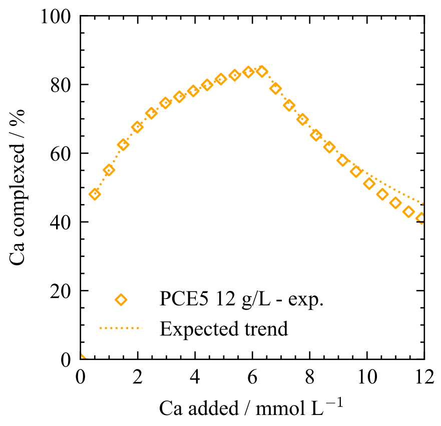

Figure S7: The percentage of calcium complexed as a function of calcium added in the CH nucleation experiments in solutions containing 12 g L^{-1} of PCE5. The symbols are for the direct measurement, while the line is the expected trend from the maximal calcium capacity of the polymers.

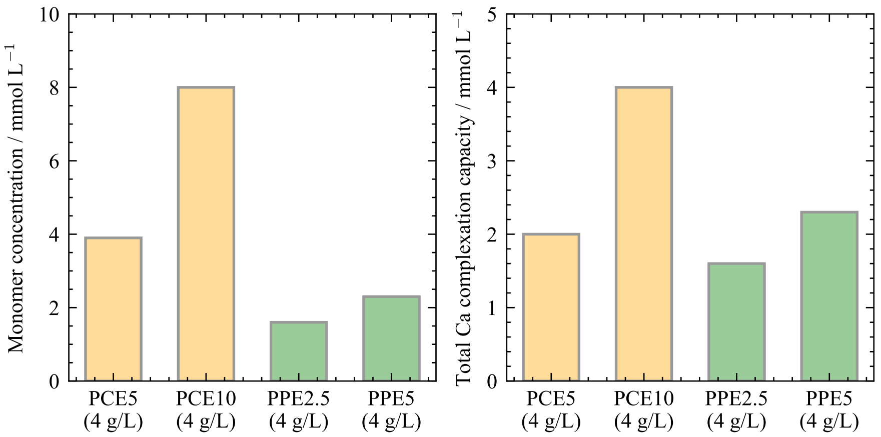

Figure S8: (A) The concentration (in $\mathrm{mmol\,L^{-1}}$) of monomer (i.e., negatively-charged functional groups), and (B) the total calcium complexation capacity of a polymer solution with a polymer concentration of $4\,\mathrm{g\,L^{-1}}$.

S3.2 Dissolution rate fit

As discussed in the main manuscript, the experimental Si and Ca concentration evolution as a function of time was fitted using equation ?? (Figure S9). The fitted Si and Ca concentrations at equilibrium are observed to converge toward the solubility of C-S-H Figure S10). The dissolution rates shown in the manuscript where determined at Ca and Si concentration ranges well below the solubility limit of C-S-H as determined by Kulik et al. [9] and by Haas and Nonat [10] (Figure S11).

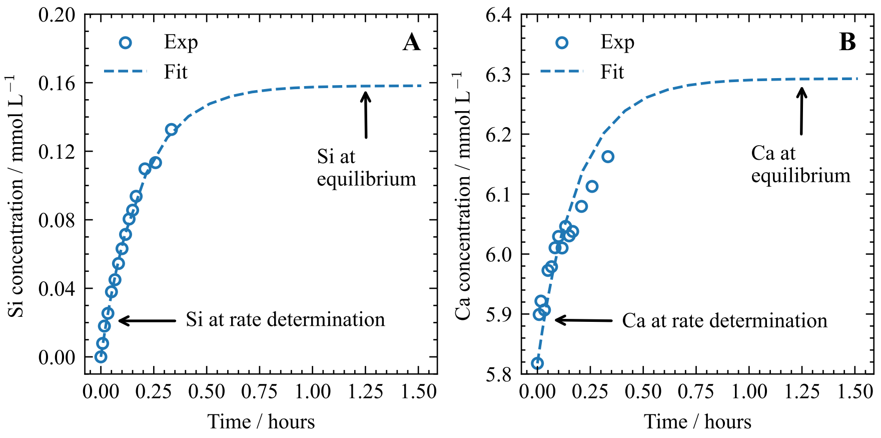

Figure S9: Schematic illustration of the time points for the ionic concentrations at rate determination and at equilibrium for A) Silicon and B) Calcium using the example of the reference system in the presence of $6\,\mathrm{mmol\,L^{-1}}$ of $Ca(OH)_2$.

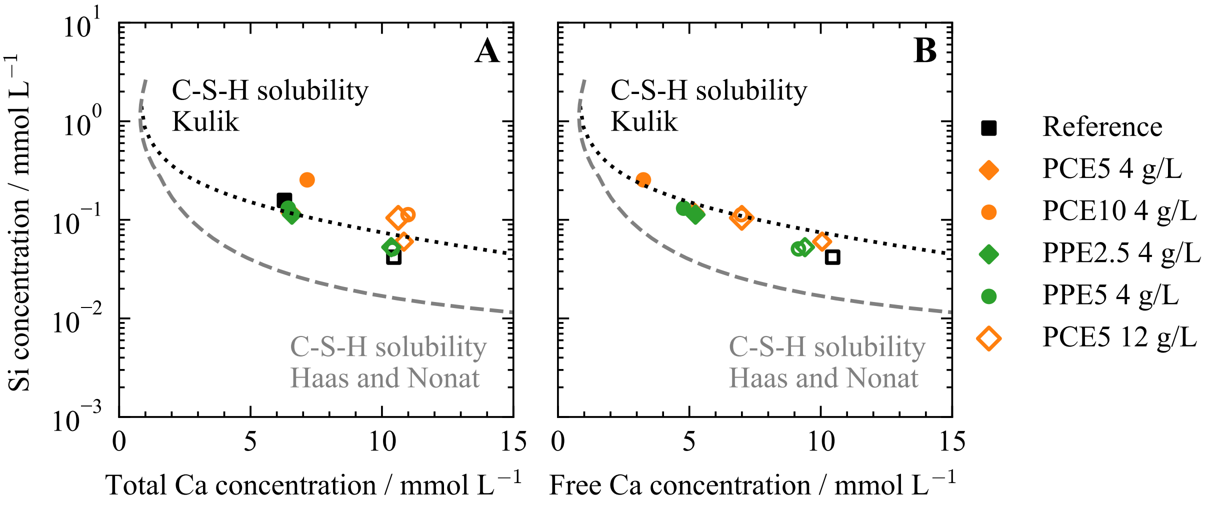

Figure S10: Equilibrium concentrations of silicon in solution for A) the total calcium concentration in solution and B) the free calcium concentration (i.e., only the non-complexed calcium in solution). For reference, the modeled solubility limit of C-S-H determined by Kulik [9] (black dotted line) and by Haas and Nonat [10] (grey dashed lined) was added to the plots.

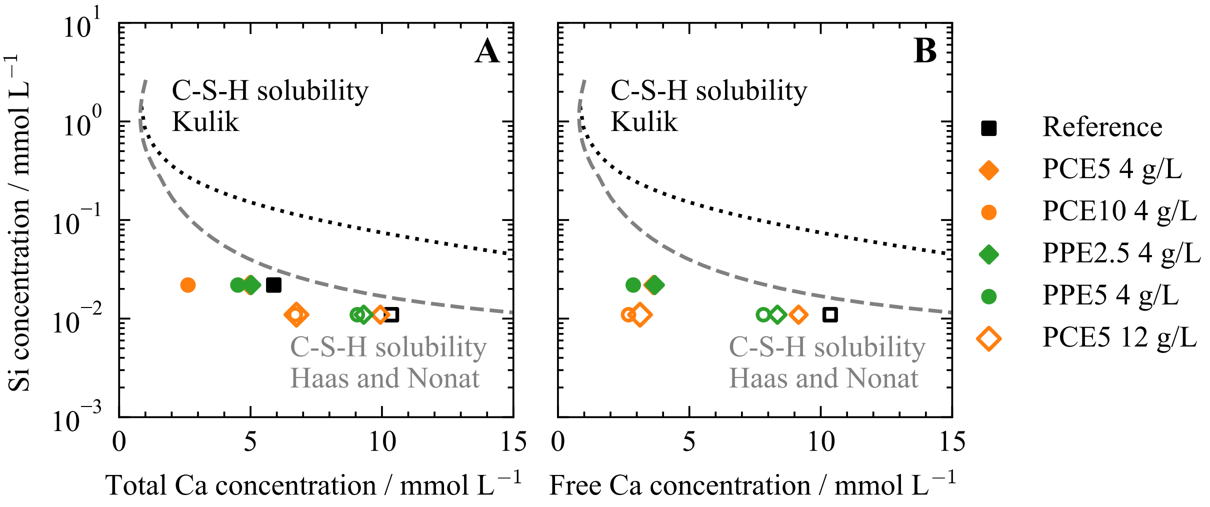

Figure S11: Silicon concentrations at rate determination for A) the total calcium concentration in solution and B) the free calcium concentration (i.e., only the non-complexed calcium in solution). For reference, the modeled solubility limit of C-S-H determined by Kulik [9] (black dotted line) and by Haas and Nonat [10] (grey dashed lined) was added to the plots.

In general, the dissolution rates measured in $Ca(OH)_2$-containing solutions follows a sigmoidal trend as a function of $\ln(\Pi)$ that can be fitted using the following function:

$$-r = \frac{-r_{max}}{2} \cdot \left[1 - \mathrm{erf}\,\frac{\ln(\Pi) - \ln(\Pi)_0}{\ln(\Pi)_1 - \ln(\Pi)_0}\right] \tag{S1}$$

where the rates are expressed in $\mu\mathrm{mol\,m^{-2}\,s^{-1}}$, r_{max} is the average dissolution rate in high undersaturation conditions (i.e., when $\ln(\Pi)$ is below -80), $\ln(\Pi)_0$ is the $\ln(\Pi)$ value at the inflection point, and $\ln(\Pi)_1$ is the $\ln(\Pi)$ value at which the dissolution rate r_1 satisfies the following:

$$-r_1 = \frac{-r_{max}}{2} \cdot [1 - \text{erf}\, 1] \qquad \text{(S2)}$$

When fitting seven distinct datasets sourced from the literature with equation S1 [11–13], we found that $\ln(\Pi)_0$ and $\ln(\Pi)_1$ showed minimal variation. Consequently, we fitted a total of 195 data points collected from the literature, yielding values of $\ln\Pi_0 = -69.3$ and $\ln\Pi_1 = -62.6$, respectively. In contrast, r_{max} values were observed to vary significantly. That is because variations in experimental conditions (e.g., pellet dissolution vs. batch powder dissolution, BET vs. geometric surface normalization, flow rate in flow-through experiments, etc.) can induce variation in the measured rates up to one order of magnitude [14, 15]. The fitted r_{max} for each dataset sourced from the literature are provided in Table S3. The value used here ($107.0\,\mu\text{mol}\,\text{m}^{-2}\,\text{s}^{-1}$) is an average of the values fitted from the three datasets of Nicoleau et al. [11]. Various dissolution kinetic models [11, 16–18] and deep learning models [19] have been proposed to estimate the dissolution rate of C_3S in an extensive range of conditions. The fit function proposed here is not meant to have an intrinsic physical meaning. Still, it is proposed as a simple tool to facilitate dataset comparison across various experimental conditions. For example, in the present work, all dissolution rates sourced from Marchon et al. [20] were multiplied by a factor of 2.87, which corresponds to the variation in r_{max} compared to the datasets from Nicoleau et al. [11], allowing for a better comparison across a large range of ln(Π) values (Figure S12). Note that the choice to normalize the rates of Marchon et al. to align with the dataset of Nicoleau et al. [11] is arbitrary and does not reflect any judgment on the accuracy of the original values in either dataset.

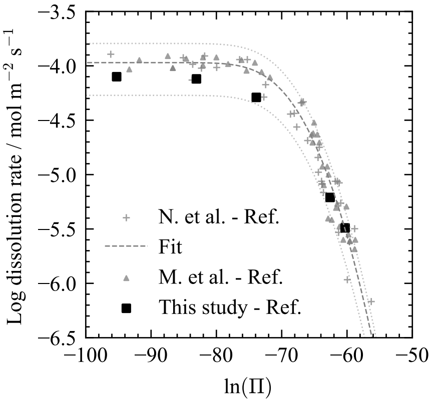

Figure S12: Comparison between the dissolution rate measured by Marchon et al. [13] multiplied by a factor of 2.87, the dissolution rate measured in this study, and the dissolution rate measured by Nicoleau et al. [11], plotted as a function of ln(Π). All rates were measured in $Ca(OH)_2$-containing solutions devoid of additives. The fitted dashed line was obtained using equation 6 in results section 3.1. The dotted lines represent the 50 % confidence interval.

Table S3: Fit parameters obtained by fitting multiple datasets sourced from the literature [11–13]. For the dataset of Nicoleau et al. [11], the $\ln(\Pi)_1$ was first recalculated using the CEMDATA18 database [21] that takes into account aqueous calcium silicate complexes. (1) Parameters used for the fit presented in this study.

	C_3S type	Dissolution conditions	$\ln(\Pi)_0$	$\ln(\Pi)_1$	r_{max} (µmol m^{-2} s^{-1})	r_{ref}/r_{max}	R^2	MAPE (%)
Nicoleau et al.	Average	Batch powder dissolution	-69.3 (1)	-62.6 (1)	107 (1)	1 (ref)	0.920	12.6
	$C_3S - t1$				123.3	0.87	0.963	35.7
	$C_3S - t2$				99.2	1.08	0.997	38.9
	$C_3S - m$				74	1.45	0.992	33.2
Juilland et al.	$C_3S - m$	Flow-through-cell	-69.3 (1)	-62.6 (1)				
		35 mL/min/mm^2			45.6	2.35	0.965	22.8
		18 mL/min/mm^2			36	2.98	0.982	18.6
		9 mL/min/mm^2			28.3	3.79	0.962	18.5
Marchon et al.	$C_3S - m$	18 mL/min/mm^2	-69.3 (1)	-62.6 (1)	37.4	2.87	0.978	22.0

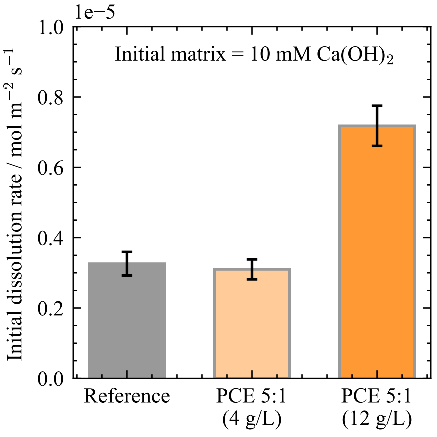

Figure S13: Additional dissolution experiments with 4 and 12 g L^{-1} PCE5 at an initial $Ca(OH)_2$ concentration of 12 mmol L^{-1}.

Table S4: Extended table for the dissolution experiments with different starting conditions. Equal conditions of temperature T = 20 °C, and water/solid ratio = 10 000 were chosen. The R^2 and MAPE values in bracket were determined by excluding an outlier. CH stands for $Ca(OH)_2$.

Starting conditions	Amount dissolved at rate estimation (%)	Element concentrations at rate estimation: Free Ca ($mmol\,L^{-1}$)	Complexed Ca ($mmol\,L^{-1}$)	Si ($mmol\,L^{-1}$)	Na ($mmol\,L^{-1}$)	Ion activities at rate estimation: (OH^-)	(Ca^{2+})	(H_4SiO_4)	ln(Π)	Goodness of fit for rate estimation: R^2 (%)	MAPE ($mol\,m^{-2}\,s^{-1}$)	Dissolution rate normalized to GSA
DIW	5	0.07	0	0.22×10^{-1}	0	0.12×10^{-3}	0.61×10^{-4}	0.78×10^{-5}	−95.3	0.939 [0.967]	11.62 [6.48]	8×10^{-5}
DIW	20	0.26	0	0.88×10^{-1}	0	0.43×10^{-3}	2.29×10^{-4}	1.08×10^{-5}	−83.1	0.939 [0.967]	11.62 [6.48]	7.6×10^{-5}
DIW	60	0.79	0	2.63×10^{-1}	0	1.22×10^{-3}	6.06×10^{-4}	1.1×10^{-5}	−73.9	0.939 [0.967]	11.62 [6.48]	6×10^{-5}
6 mM CH	5	5.88	0	2.19×10^{-2}	0	1.01×10^{-2}	3.21×10^{-3}	1.93×10^{-8}	−62.6	0.997	5.14	6.2×10^{-6}
10 mM CH	2.5	10.36	0	1.09×10^{-2}	0	1.7×10^{-2}	4.77×10^{-3}	2.52×10^{-9}	−60.3	0.955	11.06	3.3×10^{-6}
6 mM CH + 4 g/L PCE5	5	4.97	1.34	2.19×10^{-2}	0	8.57×10^{-3}	2.83×10^{-3}	2.86×10^{-8}	−63.5	0.983	11.2	7.7×10^{-6}
10 mM CH + 4 g/L PCE5	2.5	9.94	0.78	1.09×10^{-2}	0	1.63×10^{-2}	4.64×10^{-3}	2.79×10^{-9}	−60.5	0.987	9.86	3.1×10^{-6}
10 mM CH + 12 g/L PCE5	2.5	6.75	3.62	1.09×10^{-2}	0	1.14×10^{-2}	3.55×10^{-3}	6.98×10^{-9}	−62.5	0.988	7.42	7.2×10^{-6}
6 mM CH + 4 g/L PCE10	5	2.62	3.9	2.19×10^{-2}	0	4.69×10^{-3}	1.73×10^{-3}	11.27×10^{-8}	−67.3	0.989	8.98	2×10^{-5}
10 mM CH + 4 g/L PCE10	2.5	6.7	4.02	1.09×10^{-2}	0	1.14×10^{-2}	3.53×10^{-3}	7.09×10^{-9}	−62.6	0.991	9.01	6.9×10^{-6}
6 mM CH + 4 g/L PPE2.5	5	5.03	1.34	2.19×10^{-2}	3.3	1.15×10^{-2}	2.68×10^{-3}	1.76×10^{-8}	−62.4	0.986	11.32	6.1×10^{-6}
10 mM CH + 4 g/L PPE2.5	2.5	9.31	0.95	1.09×10^{-2}	3.3	1.81×10^{-2}	4.23×10^{-3}	2.5×10^{-9}	−60.3	0.966	14.44	3.1×10^{-6}
6 mM CH + 4 g/L PPE5	5	4.52	1.65	2.19×10^{-2}	4.6	1.18×10^{-2}	2.4×10^{-3}	1.84×10^{-8}	−62.6	0.982	12.55	7.1×10^{-6}
10 mM CH + 4 g/L PPE5	2.5	9.06	1.24	1.09×10^{-2}	4.6	1.88×10^{-2}	4.08×10^{-3}	2.4×10^{-9}	−60.2	0.985	10.52	3×10^{-6}

S4 Nucleation

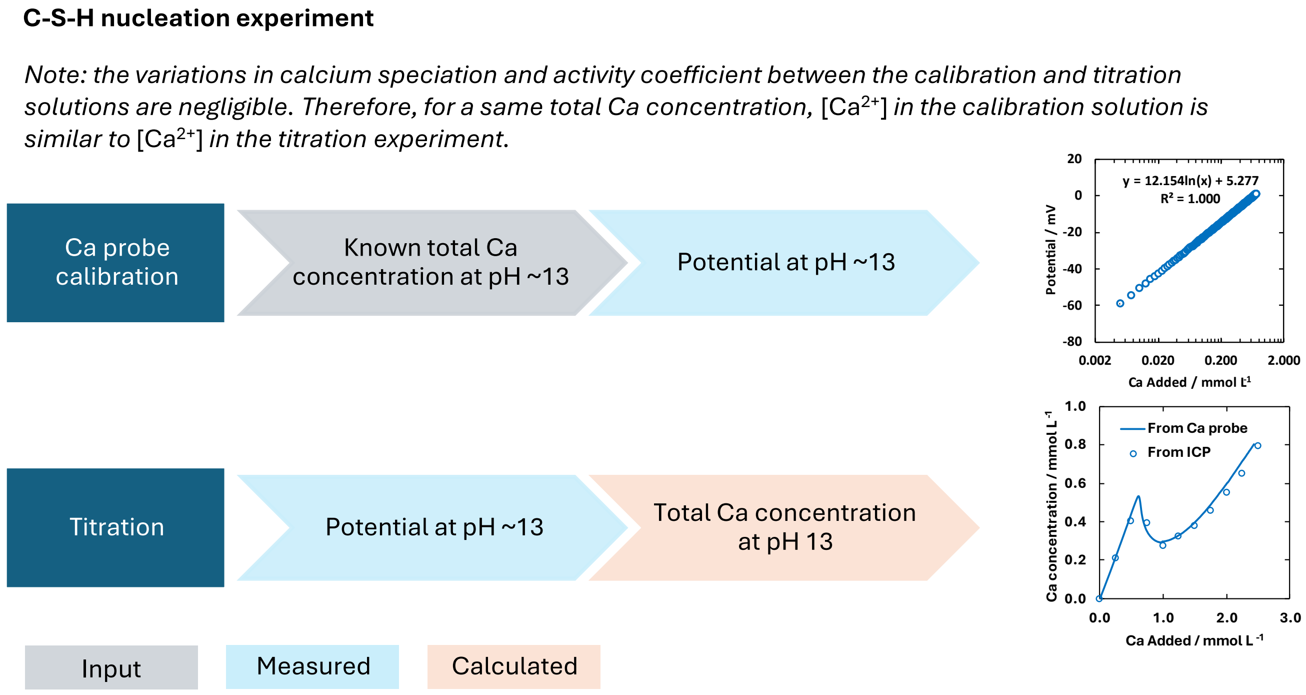

Figure S14: Schematic representation of the calibration method used to acquire calcium concentration curves during the C-S-H titration experiments. This method is supported by the good agreement observed between the calculated curve and the punctual calcium concentration measurement by ICP.

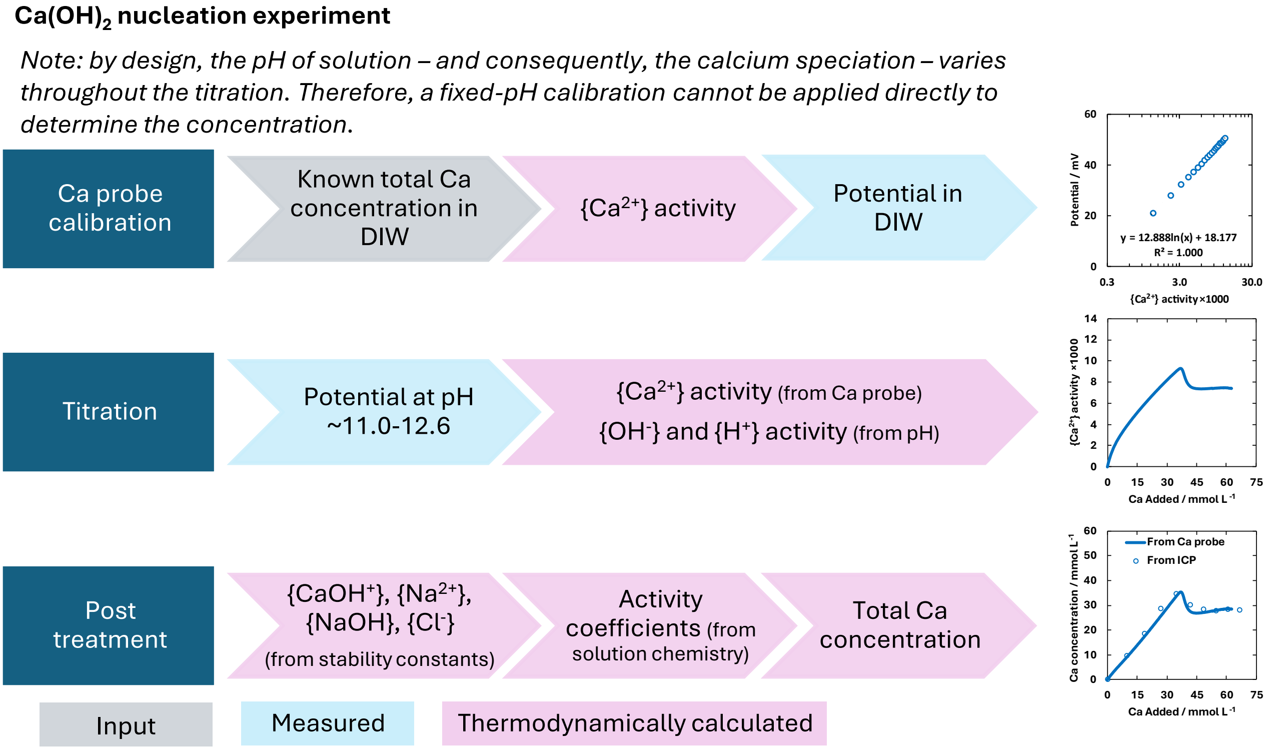

Figure S15: Schematic representation of the calibration method used to acquire calcium concentration curves during the portlandite titration experiments. This method is supported by the good agreement observed between the calculated curve and the punctual calcium concentration measurement by ICP.

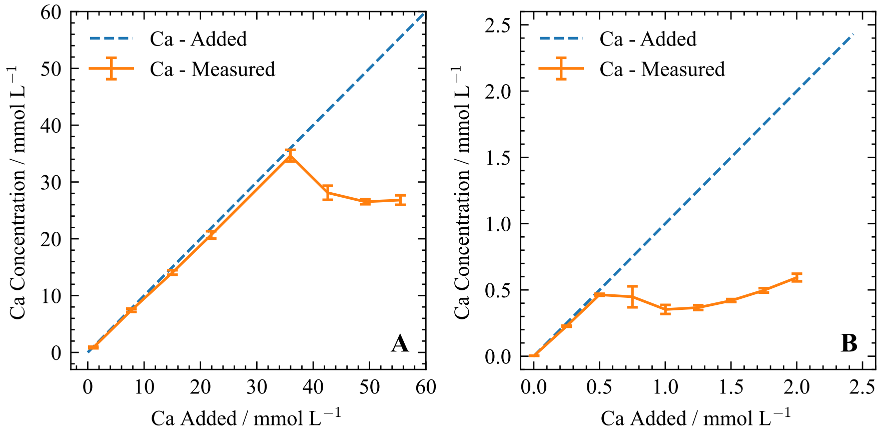

Figure S16: Mean values and error bars of three individual measurements of A) Portlandite and B) C-S-H nucleation.

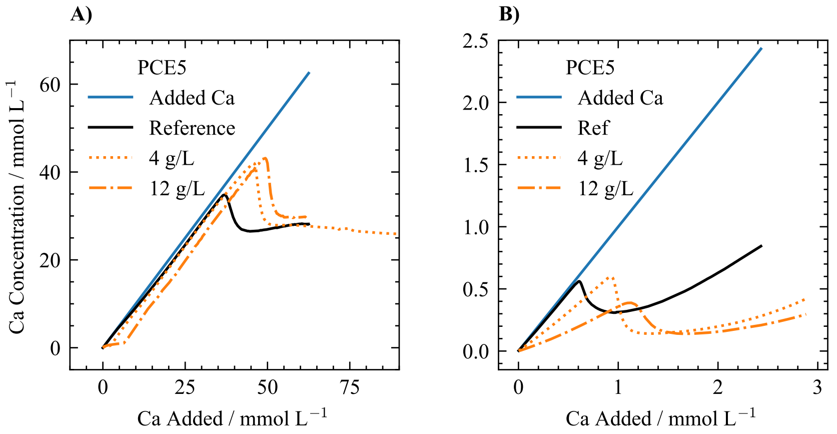

Figure S17: Additional nucleation experiments with 4 and 12 g L^{-1} PCE5 of A) Portlandite and B) C−S−H.

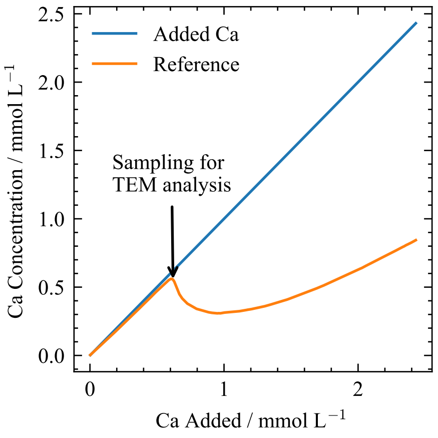

Figure S18: Samples for TEM analysis of the C-S-H nucleation experiments were taken at the local maximum of the nucleation curve. The black arrow shows the time point of sampling in the case of the reference experiment.

References

[1] O. Rindle, T. Gädt, “Along the S-Curve – How Superplasticizers Affect the Yield Stress of Cement Paste”, *ce/papers* **2023**, *6*, 614–620, DOI 10.1002/cepa.2914.

[2] O. Rindle, T. Gaedt, “The Purer the Better: How Monomer Purity Affects the Effectiveness of Phosphate Type Superplasticizers in Cement Paste”, *Thailand Concrete Association Ed.* **2023**, *Further Reduction of CO2 -Emissions and Circularity in the Cement and Concrete Industry, 16th International Congress on the Chemistry of Cement 2023 - ICCC2023 (Bangkok 18.-22.09.2023)*, 73–76.

[3] P. G. Daniele, C. D. Stefano, M. Ginepro, S. Sammartano, “Salt Effects on the Protonation of Polymethacrylate and Naq, Kq, Ca2q Complex Formation”, *Fluid Phase Equilibria* **1999**.

[4] R. Jastrzab, L. Lomozik, “Stability and Coordination Mode of Complexes of Polyphosphates and Polymetaphosphates with Copper(II) Ions in Aqueous Solution—Potentiometric, Spectral and Theoretical Studies”, *Journal of Solution Chemistry* **2010**, *39*, 909–919, DOI 10.1007/s10953-010-9558-1.

[5] X. Li, A. Ouzia, K. Scrivener, “Laboratory Synthesis of C3S on the Kilogram Scale”, *Cement and Concrete Research* **2018**, *108*, 201–207, DOI 10.1016/j.cemconres.2018.03.019.

[6] A. Vohburger, M. Collin, A. Bouissonnié, T. Gädt, “Citric, Tartaric, and Succinic Acid Effects on C3S Dissolution and the Nucleation Kinetics of C-S-H and Portlandite”, *ChemRxiv* **2024**, DOI 10.26434/chemrxiv-2024-6pk1dDOI:10.26434/chemrxiv-2024-6pk1d.

[7] A. Vohburger, T. Gädt, “Influence of Carboxylic Acids on the Nucleation of Cementitious Phases Studied by Titrimetric Methods”, *ce/papers* **2023**, *6*, 22–29, DOI 10.1002/cepa.2891.

[8] D. Marchon, Doctoral Thesis, ETH Zurich, Zurich, **2016**, DOI 10.3929/ethz-a-010798278.

[9] D. A. Kulik, “Improving the Structural Consistency of C-S-H Solid Solution Thermodynamic Models”, *Cement and Concrete Research* **2011**, *41*, 477–495, DOI 10.1016/j.cemconres.2011.01.012.

[10] J. Haas, A. Nonat, “From C–S–H to C–A–S–H: Experimental Study and Thermodynamic Modelling”, *Cement and Concrete Research* **2015**, *68*, 124–138, DOI 10.1016/j.cemconres.2014.10.020.

[11] L. Nicoleau, A. Nonat, D. Perrey, “The Di- and Tricalcium Silicate Dissolutions”, *Cement and Concrete Research* **2013**, *47*, 14–30, DOI 10.1016/j.cemconres.2013.01.017.

[12] P. Juilland, E. Gallucci, “Morpho-Topological Investigation of the Mechanisms and Kinetic Regimes of Alite Dissolution”, *Cement and Concrete Research* **2015**, *76*, 180–191, DOI 10.1016/j.cemconres.2015.06.001.

[13] D. Marchon, P. Juilland, E. Gallucci, L. Frunz, R. J. Flatt, “Molecular and Submolecular Scale Effects of Comb-Copolymers on Tri-Calcium Silicate Reactivity: Toward Molecular Design”, *Journal of the American Ceramic Society* **2017**, *100*, 817–841, DOI 10.1111/jace.14695.

[14] M. Fournier, A. Ull, E. Nicoleau, Y. Inagaki, M. Odorico, P. Frugier, S. Gin, “Glass Dissolution Rate Measurement and Calculation Revisited”, *Journal of Nuclear Materials* **2016**, *476*, 140–154, DOI 10.1016/j.jnucmat.2016.04.028.

[15] R. S. Arvidson, I. E. Ertan, J. E. Amonette, A. Luttge, “Variation in Calcite Dissolution Rates:” *Geochimica et Cosmochimica Acta* **2003**, *67*, 1623–1634, DOI 10.1016/S0016-7037(02)01177-8.

[16] V. Robin, B. Wild, D. Daval, M. Pollet-Villard, A. Nonat, L. Nicoleau, “Experimental Study and Numerical Simulation of the Dissolution Anisotropy of Tricalcium Silicate”, *Chemical Geology* **2018**, *497*, 64–73, DOI 10.1016/j.chemgeo.2018.08.023.

[17] J. W. Bullard, G. W. Scherer, J. J. Thomas, “Time Dependent Driving Forces and the Kinetics of Tricalcium Silicate Hydration”, *Cement and Concrete Research* **2015**, *74*, 26–34, DOI 10.1016/j.cemconres.2015.03.016.

[18] P. Martin, J. J. Gaitero, X. M. Aretxabaleta, M. J. A. Qomi, H. Manzano, “A Kinetic Monte Carlo Study of the C 3 S Dissolution Mechanism”, *Cement and Concrete Research* **2024**, *180*, 107502, DOI 10.1016/j.cemconres.2024.107502.

[19] T. Han, S. A. Ponduru, A. Reka, J. Huang, G. Sant, A. Kumar, “Predicting Dissolution Kinetics of Tricalcium Silicate Using Deep Learning and Analytical Models”, *Algorithms* **2022**, *16*, 7, DOI 10.3390/a16010007.

[20] D. Marchon, U. Sulser, A. Eberhardt, R. J. Flatt, “Molecular Design of Comb-Shaped Polycarboxylate Dispersants for Environmentally Friendly Concrete”, *Soft Matter* **2013**, *9*, 10719, DOI 10.1039/c3sm51030a.

[21] B. Lothenbach, D. A. Kulik, T. Matschei, M. Balonis, L. Baquerizo, B. Dilnesa, G. D. Miron, R. J. Myers, “Cemdata18: A Chemical Thermodynamic Database for Hydrated Portland Cements and Alkali-Activated Materials”, *Cement and Concrete Research* **2019**, 472–506, DOI 10.1016/j.cemconres.2018.04.018.
